# Local-Scale Patterns of Genetic Variability, Outcrossing, and Spatial Structure in Natural Stands of *Arabidopsis thaliana*

**DOI:** 10.1371/journal.pgen.1000890

**Published:** 2010-03-26

**Authors:** Kirsten Bomblies, Levi Yant, Roosa A. Laitinen, Sang-Tae Kim, Jesse D. Hollister, Norman Warthmann, Joffrey Fitz, Detlef Weigel

**Affiliations:** 1 Department of Molecular Biology, Max Planck Institute for Developmental Biology, Tübingen, Germany; 2 Department of Organismic and Evolutionary Biology, Harvard University, Cambridge, Massachusetts, United States of America; University of Georgia, United States of America

## Abstract

As *Arabidopsis thaliana* is increasingly employed in evolutionary and ecological studies, it is essential to understand patterns of natural genetic variation and the forces that shape them. Previous work focusing mostly on global and regional scales has demonstrated the importance of historical events such as long-distance migration and colonization. Far less is known about the role of contemporary factors or environmental heterogeneity in generating diversity patterns at local scales. We sampled 1,005 individuals from 77 closely spaced stands in diverse settings around Tübingen, Germany. A set of 436 SNP markers was used to characterize genome-wide patterns of relatedness and recombination. Neighboring genotypes often shared mosaic blocks of alternating marker identity and divergence. We detected recent outcrossing as well as stretches of residual heterozygosity in largely homozygous recombinants. As has been observed for several other selfing species, there was considerable heterogeneity among sites in diversity and outcrossing, with rural stands exhibiting greater diversity and heterozygosity than urban stands. Fine-scale spatial structure was evident as well. Within stands, spatial structure correlated negatively with observed heterozygosity, suggesting that the high homozygosity of natural *A. thaliana* may be partially attributable to nearest-neighbor mating of related individuals. The large number of markers and extensive local sampling employed here afforded unusual power to characterize local genetic patterns. Contemporary processes such as ongoing outcrossing play an important role in determining distribution of genetic diversity at this scale. Local “outcrossing hotspots” appear to reshuffle genetic information at surprising rates, while other stands contribute comparatively little. Our findings have important implications for sampling and interpreting diversity among *A. thaliana* accessions.

## Author Summary

The popular model plant *Arabidopsis thaliana* is increasingly used to investigate questions in evolution and ecology. Thus it is important to understand the dynamics of wild populations at a scale relevant to single plants. We analyzed over 1,000 individuals from 77 ecologically diverse stands near Tübingen in Southwestern Germany. By assaying hundreds of independent markers in their genomes, we generated an unprecedentedly detailed view of local relatedness and recombination patterns. As has been observed previously for *Arabidopsis thaliana* and other self-compatible plants, even closely neighboring stands were strongly differentiated. Nevertheless, individuals tended to be most closely related to near neighbors, and footprints of recent recombination events were apparent. Structure was evident within stands, suggesting short dispersal ranges and the potential for nearest neighbor mating to reduce heterozygosity. We also observed differences between stands in rural and urban settings: stands in species-rich rural sites had higher average genetic diversity and presented more evidence of past and ongoing outcrossing than their species-poor urban counterparts. Thus novel combinations of genes may primarily arise in a subset of stands that act as “outcrossing hotspots,” while others contribute little to increasing genetic diversity.

## Introduction

Gaining a detailed understanding of *Arabidopsis thaliana* in its native context is becoming especially important as this species is increasingly employed as a model in studies of adaptation and evolution [1],[2]. *Arabidopsis thaliana* is an annual herb that exists in the wild in fragmented populations throughout much of the northern hemisphere. It is self-compatible and wild populations are highly homozygous – average outcrossing rates have been estimated in the range of 0.3 to 2.5% [e.g., [3]–[6]].

A large body of literature on the population genetics of self-fertilizing plants established already decades ago that self-fertilizing species often exhibit strong local differentiation of individual stands and that stands are often not genetically homogeneous [e.g., [7]–[15]]. Numerous studies published since have also demonstrated a tendency for high heterogeneity in measures of genetic diversity and heterozygosity among stands [9],[16]. This pattern has been observed many times and is generally stronger in self-fertilizing than outcrossing species [16]. Differences in diversity or heterozygosity that correlated with specific habitat characteristics have been documented in several systems, one example being higher outcrossing in mesic than xeric sites [e.g., [7],[8],[15]].

Genetic variation in *A. thaliana* follows the same basic patterns as other self-fertilizing species, but the molecular resources and extensive sampling available in *A. thaliana* have allowed a much more fine-grained analysis of these patterns. Like other selfers, *A. thaliana* does not exist exclusively in monotypic stands, and it is not completely selfing in the wild [e.g., [6], [17]–[20]]. Nevertheless, even neighboring stands are often strongly differentiated, suggesting low inter-population migration rates and limited dispersal distances [e.g., [4],[21],[22]]. Several studies have uncovered considerable variability among stands in genetic diversity and/or heterozygosity [e.g., [19], [21]–[23]]. The observation that at least some wild *A. thaliana* stands may be quite transient supports the idea that rapid turnover could contribute to patterns of strong local differentiation and high prevalence of genetically depauperate stands [21]. However, this would be complicated by the presence of a seed bank, which could buffer the effects of population turnover [24].

Population genetic patterns of *A. thaliana* have been investigated at varying geographic scales [25]. Several recent studies have provided evidence of range-wide population structure [e.g., [18], [26]–[28]], indicative of historical processes such as recolonization from different ice-age refugia, or opportunities that appeared with the spread of human agriculture [26],[28]. These results are also consistent with the view that contemporary gene flow and migration are sufficiently low, at least at large geographic scales, to give rise to an overall pattern of isolation by distance [18],[27],[29],[30]. Nevertheless, linkage disequilibrium (LD) in *A. thaliana* is generally quite low, indicating that recombination, even if rare, is sufficient to break nonrandom allele associations at a species-wide level [31],[32]. A similar trend has been observed in wild barley [33]. Extensive chromosomal stretches of haplotype identity in some pairwise comparisons within regions indicate that outcrossing among local types generates genetic novelty in *A. thaliana* by recombining pre-existing haplotypes [18]. Local populations can be strongly differentiated even when they are geographically close [4],[21],[22] and variability in diversity has been found among stands [5],[6],[20],[22]. Despite considerable advances in knowledge of local populations, few studies have sampled extensively from adjacent sites, and none of the previous studies has included a network of numerous local stands to specifically examine micro-geographic genetic structure.

Compared to our understanding of larger-scale patterns, we know much less about how contemporary processes such as outcrossing impact local population structure in *A. thaliana*. Furthermore, few studies have addressed how heterogeneous environments might affect genetic patterns at a fine geographic scale in this species. Such information is a crucial prerequisite for studies of local adaptation, and it is particularly important in view of the resources that are being invested in using *A. thaliana* for genome-wide association studies [31] and large-scale sequencing efforts [34].

We examined local-scale population genetic patterns in 77 *A. thaliana* stands distributed in a restricted region around Tübingen, in Southwestern Germany. We sampled over one thousand individuals from stands varying in size and ecological setting, and genotyped progeny with 436 intermediate-frequency single nucleotide polymorphism (SNP) markers distributed across the genome [35]. This large number of markers and extensive local sampling provided a uniquely detailed picture of patterns of relatedness and heterozygosity, and the scale at which these patterns are evident in the landscape. Finally, we revisited several stands one year later, to address how replicable the sampling would be and whether similar genotypes persist within local stands over multiple years, or whether migration or germination from seed banks might infuse novel variation.

## Results

### Local Tübingen stands

**Figure 1:**
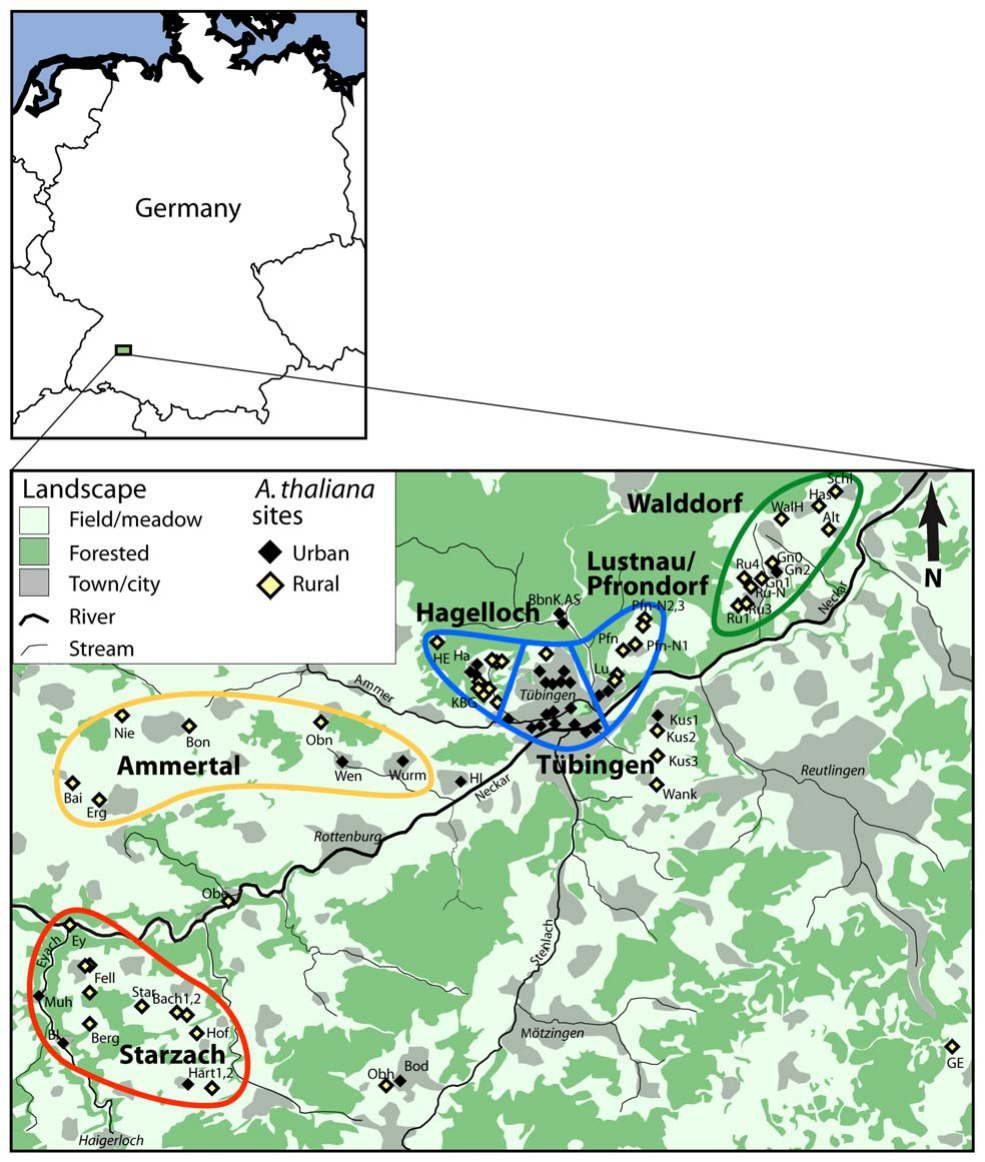
Map of collection sites in Tübingen area. Sub-region names are indicated and color-coded to match colors used in cluster diagrams in Figure 3 and Figure 5.

From April to June 2007, we sampled *A. thaliana* within an area comprising approximately 460 square kilometers in the Neckar river valley around the town of Tübingen in Southwestern Germany (Figure 1, Table S1). We collected seeds from 1,005 individuals from 77 stands. We defined a stand as a single cluster of plants separated from other groups by at least 35 meters. This threshold was used because it was the lowest distance that we observed between clearly distinct groups without any intervening plants. Though it is possible in some cases for pollen of selfing plants to travel further than this distance [e.g., [36]], we observed very strong differentiation among most neighboring stands, even when they were very closely spaced, and thus kept them separate in further analyses. We refer to the physical locations of stands as “sites.” Across the entire region, pairwise physical distance between sampled stands ranged from 35 m to 40 km, with the most isolated stand being 16 km from its closest sampled neighbor. The average distance of stands to their closest sampled neighbor was 1.7 km. Stands varied considerably in size, from one or a few individuals to thousands of plants. Where stands consisted of fewer than 20 individuals, we sampled all plants present. For stands larger than 20, we sampled 20 to 30 individuals.

The individual collection sites had a range of different physical characteristics, and covered examples with high human impact in urban settings, as well as sites in rural environments in meadows and field borders with less ongoing human influence. In meadow sites, the presence of *A. thaliana* plants was often associated with vole or mole activity, suggesting that the small mounds of upheaved or cleared earth produced by these animals provide sufficient disturbed ground to support *A. thaliana* in otherwise highly competitive meadow environments.

Stands varied considerably in the number of genotypes found. Twenty-three of the 77 stands (30%) were monotypic, that is all individuals sampled were identical at all 436 markers. The remaining 56 stands each contained two or more distinct types. While there was a general trend for stands with only a single genotype to be smaller than stands with two or more genotypes (average 12.2 vs. 17.3 individuals; p = 0.047), some larger stands were also monotypic and many small stands contained multiple genotypes (Table S1). Among stands with ten or more individuals, 18% were monotypic, and of those with 20 or more plants, 15% were monotypic. Stands with multiple genotypes differed along a continuum in the prevalence of each of the distinct types: at the extremes, some stands were dominated by one or a few common genotypes, while others were made up of many rare genotypes (Table S1, Table S2). Consistent with this, there was considerable variation among sites for genetic diversity (see below). Overall, we identified 324 unique multi-locus genotypes, of which 247 were fully homozygous.

### Diversity and heterozygosity

Since naturally occurring *A. thaliana* stands varied considerably in size, we asked whether this might affect genetic diversity or observed heterozygosity. Unsurprisingly, the number of plants sampled in a stand correlated significantly with the number of distinct genotypes identified (correlation = 0.46, p = 0.0002, r^2^ = 0.21). However, several other parameters were not strongly correlated with stand size, including genetic diversity measured as *H*_e_ (r = 0.166, p = 0.21) or 1-Q (r = 0.044, p = 0.74). Even correlation with observed heterozygosity was weak (r = −0.26, p = 0.085). Any trends were primarily due to smaller stands: For 39 stands containing ten or more individuals, the relationship between stand size and *H*_e_ (r = 0.026, p = 0.870), 1-Q (r = 0.04, p = 0.822), and observed heterozygosity (r = −0.14, p = 0.424) were very weak. Therefore, for further analyses of stand diversity and heterozygosity, we used only this subset.

Despite the lack of strong correlations between stand size and population parameters, we could not exclude that sample size differences could affect estimates of diversity and heterozygosity [37]. Thus, in order to make genetic parameters of populations more directly comparable and to compensate for variation in sample sizes, we employed a sub-sampling approach (see Materials and Methods; Table S3).

Both *H*_e_ and the inbreeding statistic *F*_IS_ were variable among populations. *H*_e_ ranged from 0 for monotypic stands to 0.318 for *H*_e_ (Table S3, Figure S1). Average *F*_IS_ across the whole dataset was 0.969 (±0.0001) indicating an overall effective outcrossing rate of 1.6% for the entire Tübingen area. This is well within the range of previous estimates, which ranged from 0.3 to 2.5% [e.g., [3],[4],[6]]. The average value obscures considerable heterogeneity among stands. Most stands in our dataset (64%) had no evidence of outcrossing, whereas others had estimated effective outcrossing rates considerably higher than what has been previously reported for *A. thaliana* (Table S3, Figure S1). The TuHO stand had a particularly low *F*_IS_ (0.69) but this was due to a single outcrossed individual in a stand that had otherwise almost no diversity (Table S3). The lowest *F*_IS_ among the remaining stands was 0.75, which reflects considerable heterozygosity compared with most other stands, and translates to an estimated effective outcrossing rate of 14.5% (Table S3). High variation in diversity and heterozygosity as we observed here is consistent with what has been reported for other self-compatible species [e.g., [10],[13]]. Variation in genetic diversity has also been reported in other studies of *A. thaliana* [e.g., [19], [21]–[23]].

### Patterns of recombination and heterozygosity

Since marker heterozygosity indicated recent outcrossing, we examined the distribution of SNP differences and heterozygosity across the genome in more detail, to obtain direct evidence of recombination among resident genotypes. Our high marker density, with on average one marker per 250 kb, gave us good power to uncover footprints of past or ongoing recombination. When comparing SNP genotypes of two unrelated individuals, or related genotypes individuals descended from a common ancestor without recombination (diverging purely by mutation), allele differences should be randomly distributed across the genome. In the majority of pairwise comparisons of genotypes between stands in our dataset, this was indeed what we observed (data not shown). This was also often true of pairwise comparisons of distinct types within stands, particularly in genetically simple stands with a small number of predominant homozygous genotypes.

However, pairwise comparisons of genotypes in some stands revealed patterns of allele sharing in mosaic blocks of identical and diverged sequence (Figure S2). This pattern is suggestive of a history of outcrossing and recombination followed by self-fertilization. Indeed, in two stands, Ey and Obn, all of the numerous distinct genotypes detected at each locale could be attributed to different combinations of only two ancestral genotypes (Figure S2A). Hence, these stands were effectively natural recombinant inbred lines. Some continued gene exchange among recombined types within each stand was evident in varying degrees of heterozygosity in individuals. The existence of distinct fully homozygous recombinant genotypes suggests that these stands have been stable for numerous generations and that the descendants of ancestral outcrossing events continue to populate these sites.

**Figure 2:**
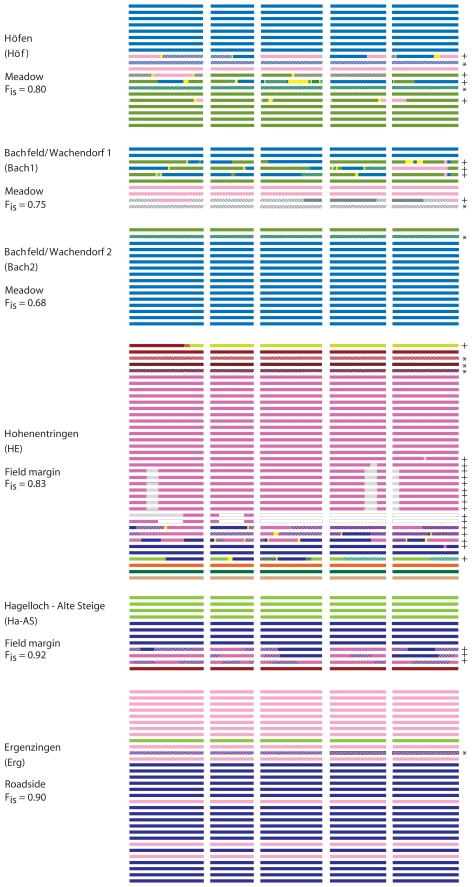
Diagram of haplotype block identity and recombination patterns in several rural stands. The columns represent the five chromosomes of *A. thaliana*, and each line represents an individual plant. Haplotypes are color-coded to indicate regions of allele identity within populations. Yellow indicates regions where putative parents were identical and recombination breakpoints were ambiguous. Plants from Ergenzingen (Erg) are shown in the order in which they were found. For other populations, individuals are ordered by similarity. “_*_” in the right hand column indicates first-generation outcrossed progeny (pollination event in spring 2007). “+” in the right hand column indicates a later-generation outcrossed descendant or homozygous individual with a clearly recombinant genotype.

In addition to historical recombination and introgression events, in some stands we observed extended stretches of linked heterozygous SNPs. We found 77 such individuals (7.7% of our entire sample), which were unevenly distributed among stands. Forty-nine stands (64%) had no heterozygotes at all, while some of the remaining 28 stands had numerous heterozygotes, and others had just one or two (Table S1). In some cases putative parental genotypes were identified in the same stand, and patterns of relatedness and heterozygosity indicated both historic and ongoing genetic exchange in these stands (Figure 2).

There was some evidence of pollen flow among stands: In some cases we could not identify the pollen parent of a particular heterozygote within a sample, and in one instance we found a plant in the TüPK stand that had been pollinated by a type not detected in TüPK, but identical to one that dominated the TüV stand 75 meters away. Emphasizing the power afforded by the large number of SNPs we used, some outcrossing events would almost certainly have gone unnoticed with a smaller marker set: For example, two distinct genotypes found in the Müh stand were nearly identical, differing at only four out of the 436 SNPs, yet we found in this stand an outcrossed individual that was heterozygous for all four of these SNPs.

In some stands we found indications that spatial structure might affect the patterns of observed heterozygosity. The Erg stand, which we sampled at roughly one-meter intervals along an approximately 30 meter transect, was dominated on each side by a distinct genotype. Where the two genotype clusters met, we identified two individual progeny that were heterozygous for all SNPs differentiating the two dominant homozygous types (Figure 2, Figure S3). A similar pattern occurred in the Bai stand (Figure S3). Bai and Erg represent what may be comparatively young stands, and may be examples of an early stage in the formation of more diverse stands with mixed haplotype blocks of the sort we observed elsewhere (Figure S2).

Because in both Erg and Bai genotypes seemed to be non-randomly distributed, we examined ten other stands where samples had been collected in order. Several were spatially structured. Stands with fewer genotypes tended to show stronger clustering of identical genotypes, but even in genetically diverse stands, identical genotypes were preferentially found in close proximity to one another (Figure S3). The degree of genotype clustering, particularly the proportion of individuals flanked by two identical neighbors, was correlated with *F*_IS_, (r = 0.48; p = 0.098; Figure S3B). Though the relationship was not statistically significant at ∝ = 0.05, this trend nevertheless suggests that spatial structure within stands may impact observed heterozygosity in natural stands of *A. thaliana* (e.g., the Wahlund effect [38]).

### Local-scale differentiation, diversity, and heterozygosity

Overall, even closely spaced stands were very strongly differentiated. In only one instance were neighboring stands genetically identical: TüNR consisted of two small stands that were 120 meters apart, but together contained only a single genotype. Otherwise, very few genotypes were shared among stands: In only 15 cases did we find genotypes identical at all 436 markers in different stands (Table S4). This is compatible with low migration rates and/or failure of single multi-locus genotypes to persist for extended times. Eleven of the shared genotype pairs (73%) originated from stands that were near one another (50 meters to 1.2 kilometers apart). For example, TüKB and TüV, 220 meters apart, differed in only one rare genotype unique to the TüV stand. TüV and TüPK, 75 meters apart, shared one multi-locus genotype out of the eight present in these two stands together. The remaining four cases of individuals with identical multilocus genotypes shared between stands were found further apart, from seven to 21 kilometers, suggesting that on rare occasions longer distance dispersal occurs. Among these four cases, two involve stands (Erg and GE) located on sites with recent road construction activity, hinting at a possible human element in movement of genotypes. Though formally possible, the likelihood that identical combinations of such a large number of intermediate-frequency markers distributed across all five chromosomes could arise by processes other than maintenance of ancestral types or migration of contemporary types is extremely unlikely. Similar haplotypes that could independently form identical genotypes through anything but a very large number of recombination events were not found in this dataset. Thus we conclude that identical genotypes almost certainly arose from dispersal or from persistence of ancient types.

Many closely spaced stands, some as little as 35 meters apart, shared no identical genotypes, suggesting that despite their proximity, these sites were probably independently colonized and have experienced little or no gene flow. For example, the stands Tü-SB25/Tü-SB30 (55 meters apart), HaP, HaP2 and Ha3 (35 to 150 meters apart), Fell2/Fell3 and KBG1/KBG2 (each 110 meters apart) and Bach1/Bach2 (260 meters apart) did not share any multi-locus genotype. The few neighboring stands that did share whole-genome genotypes were all located in urban areas where dispersal by forces such as wind or tracking by humans may be more common than in more heavily vegetated rural areas.

Genetic differentiation between stands can be quantified by the fixation index, *F*_ST_. Within the Tübingen region, pairwise *F*_ST_ values among single stands of *A. thaliana* were very high, suggesting strong stand subdivision, with an average *F*_ST_ of 0.61. Though smaller stands were more likely to consist of single genotypes, high pairwise *F*_ST_ values were not solely attributable to inclusion of these sites. In a subset of 25 stands that had at least three distinct multi-locus genotypes and consisted of 10 or more sampled individuals, pairwise *F*_ST_ values still averaged 0.60. A subset of 13 populations having more than 25 individuals each had an average pairwise *F*_ST_ of 0.52. Thus even large stands with many genotypes were strongly differentiated.

### Geographic distribution of genotypes

There was no evidence of an overall pattern of isolation by distance in the Tübingen area as indicated by a Mantel test [39] (p = 0.76). We also tested for spatial autocorrelation [e.g., [40],[41]]. In an analysis of either 10 (each 3.8 km) or 30 (each 0.5 km) geographic distance classes, Moran's I [41]–[43] indicated significantly positive autocorrelation for the shortest distance classes (0–3.8 km; Figure S4A). Genetic distance, *D_G_* [44], showed a similar trend (Figure S4B). With distance bins of 0.5 km the first seven bins (up to 3.5 km) showed significant autocorrelation with Moran's I (data not shown). Not surprisingly, Ripley's aggregation index R [41] indicated that the sample overall represented a significantly clumped distribution of genotypes (0.10). This pattern of strong autocorrelation in the smallest distance classes is seen in the majority of plant species and this trend is particularly strong in self-fertilizing herbaceous species with gravity-dispersed seeds [45].

To examine whether distinct genotypes from the same population were more similar to each other than to those from other populations, we calculated pairwise genetic distance (SNP differentiation) for our whole dataset and divided the list into within- and between-stand comparisons. For between-stand comparisons, there was a roughly normal distribution of values centered on a mean of 0.58±0.09 (Figure S5). Within stands, however, the distribution of pairwise comparisons looked quite different: 4,500 out of 10,066 comparisons had a genetic distance of 0 (identical genotypes). The mean distance within populations was 0.20±0.2, or 0.35±0.2, if identical genotypes were excluded. Non-identical genotypes found within the same stand were thus on average much more similar to each other than genotypes sampled from different stands (Mann-Whitney U-test, p<0.0001; Figure S5).

**Figure 3:**
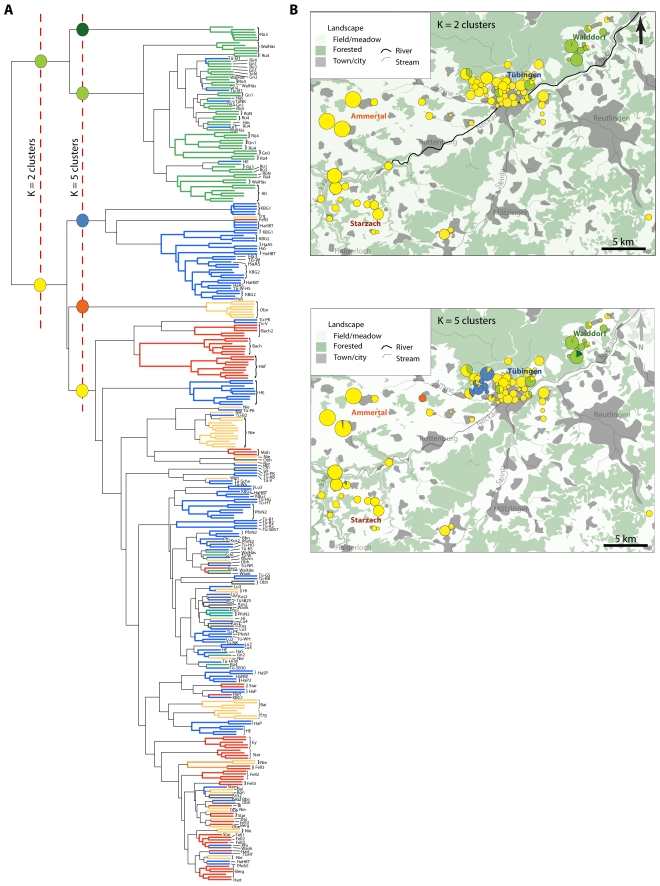
Non-parametric clustering of non-redundant Tübingen area multi-locus genotypes. (A) Cladogram of 324 non-redundant genotypes from the Tübingen area using 436 SNP markers. Branch colors indicate sub-region of origin as indicated in Figure 1. Red dotted lines indicate cutoffs for K = 2 and K = 5 clusters. Colored circles designate individual clusters. (B) Maps showing distribution of K = 2 and K = 5 clusters. Circles are approximately proportional to population size and are color-coded as indicated by the colored circles on the cladogram in (A).

A nonparametric clustering analysis, which does not rely on assumptions such as free out-crossing, revealed a tendency for genotypes from nearby stands, as well as distinct genotypes within stands, to group together (Figure 3A), though clusters from different sub-regions within the Tübingen area were often intercalated. This pattern is in agreement with previous phylogenetic analyses of local populations, where the tips of the phylogeny were clustered according to geography, but deeper nodes were not [21]. Gap statistics [46],[47] suggested two or five clusters in the Tübingen region (Figure S6A; dotted lines in Figure 3A). The distribution of genotypes belonging to each of these clusters broadly correlated with the East-West orientation that the stands followed along the Neckar river valley (Figure 3B and 3C). A major boundary was located around Tübingen, with the Eastern-most area, Walddorf (Figure 1), largely separated from the rest of the region (Figure 3). This could reflect a difference in colonization history, or that the Walddorf area is more isolated by the surrounding Schönbuch forest. Indeed, we have not found *A. thaliana* in forests around Tübingen despite repeated attempts (K.B. and L.Y., unpublished observations).

### Relationship of site type with genetic diversity and outcrossing rate

Nearly all heterozygous or obviously recombinant genotypes we observed originated from sites in rural settings, such as meadows or field borders. This prompted us to investigate more closely the relationship between site type and population genetic parameters. We classified the sites of origin as “rural” if the stands were found in meadows, near agricultural fields, or in grassy rural roadsides, and “urban” if they were in towns, where we found plants in parking areas, vacant lots, gardens, or in cracks between paving stones. To correct for sample size variation, we used only *H*_e_ and *F*_IS_ values calculated using a sub-sampling approach to compare stands.

**Figure 4:**
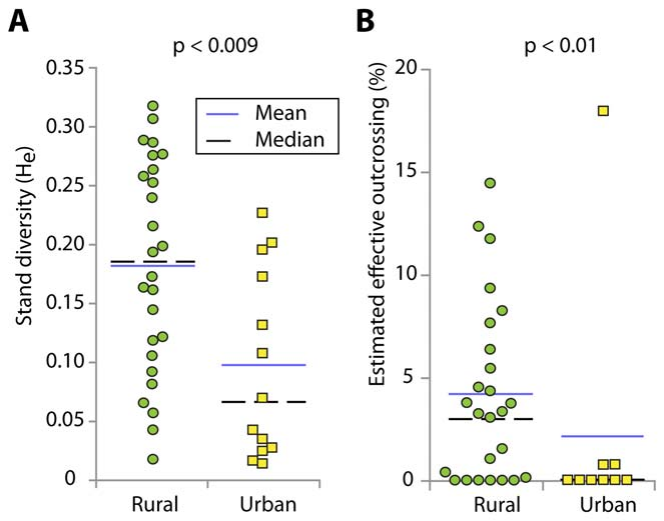
Box plots showing association of genetic diversity and effective outcrossing with site type. (A) Population genetic diversity (*H*_e_) in rural versus urban stands. (B) Estimated outcrossing (calculated from *F*_IS_) in rural versus urban stands. P-values are from Mann-Whitney U-tests comparing rural versus urban sites.

Urban stands often consisted of only a single or a few genome-wide genotype(s) while rural sites only rarely contained just a single genotype (Table S1). Urban sites had lower average genetic diversity than rural sites: Mean urban site diversity (*H*_e_) was 0.10 (95% confidence interval 0–0.26; median 0.07) while rural sites averaged 0.18 (95% confidence interval 0–0.36; median 0.18) (Figure 4, Table S3), a statistically significant difference (Mann-Whitney U-test, p<0.009).

When multiple genotypes were present in urban stands, SNP differences tended to be randomly distributed across the genome, suggesting the absence of a history of local recombination events (data not shown). Rural stands, in contrast, often showed evidence of clustering of SNP differences in pairwise genotype comparisons suggestive of historical recombination events (Figure S2). This could have resulted from differences in the prevalence of outcrossing: rural sites had significantly lower *F*_IS_ than urban sites (Mann-Whitney U-test, p<0.01). Rural sites had a mean and median *F*_IS_ of 0.92 and 0.93, respectively, while urban sites had a mean and median of 0.96 and 1.0, respectively. The mean *F*_IS_ translates to effective outcrossing of 4.1% in rural and 1.9% in urban stands, or 3.5% and 0% based on median *F*_IS_ (Figure 4). In summary, rural sites had on average higher genetic diversity as well as a higher degree of heterozygosity.

### Persistence of genotypes over time

In the spring of 2008, we returned to a subset of 21 sites that had had medium to large stands in 2007. In all of them we again found *A. thaliana* plants. We genotyped individual progeny of 369 plants with a subset of 149 markers [35], of which 133 were informative, to determine whether identical genotypes were recovered. In stands that were monotypic or genetically simple in 2007, we found mostly identical genotypes in 2008. While this is perhaps unsurprising, it does suggest that factors such as a latent genetically diverse seed bank or high migration are not contributing extensive variability from year to year at these sites. From more genetically complex stands, however, fewer identical genotypes were recovered (Table S5). In moderately diverse stands, we recovered some identical and some distinct genotypes, while in large, genetically complex meadow stands, we detected little or no genotype identity between 2007 and 2008. This suggests that these stands contained so many genotypes that our level of sampling in subsequent years was small relative to the diversity present in the entire stand. Alternatively, immigration or germination from seed banks was contributing to variation from year to year.

**Figure 5:**
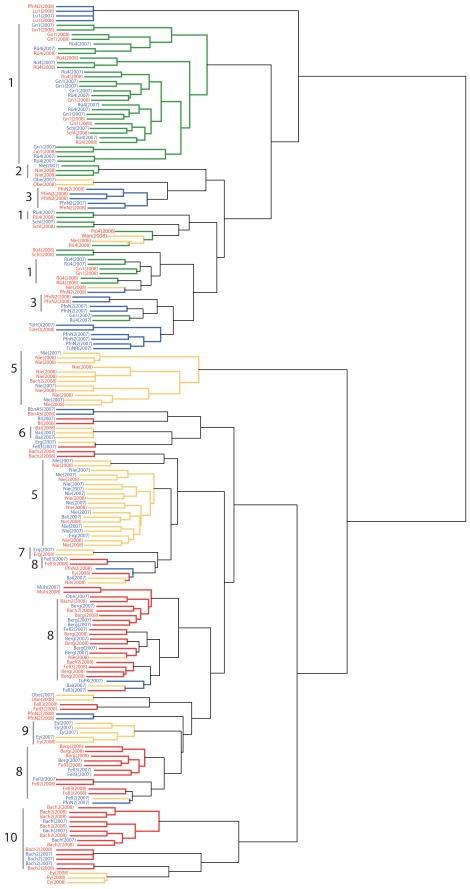
Clustering of 2007 and 2008 genotypes. Nonparametric clustering of 88 non-redundant (within stand and year) 2007 genotypes and 100 non-redundant 2008 genotypes using data from 133 SNP markers. Branches are color-coded by sub-region of origin as described for Figure 2. Samples in red are from 2008, samples in blue from 2007.

To examine whether samples in different years were effectively samples from the same larger set of genotypes, we calculated pairwise *F*_ST_ values for each site across the two years. Since sample sizes in the two years were different, we again employed a sub-sampling strategy to estimate sample differentiation among years (see Materials and Methods). Excluding stands where only a single identical genotype was found in both years, the comparisons between years gave *F*_ST_ values ranging from 0.03 to 0.13 (Table S5). That relative to between-population comparisons, *F*_ST_ values were low, but not zero, indicated that genotypes sampled in successive years were distinct, but still more closely related that genotypes sampled from different sites. This is most easily interpreted as subsamples drawn from a larger diverse population. This conclusion also supported by a cluster analysis on the 2007 and 2008 genotypes: distinct genotypes found across years tended to group together (Figure 5).

## Discussion

*Arabidopsis thaliana*, long a popular model among molecular geneticists, is increasingly being used in evolutionary and ecological research [1],[2]. To properly design and interpret evolutionary and ecological studies it is critical that we have a detailed knowledge of the population genetic patterns of natural populations of *A. thaliana*. Recognizing this need, several studies have investigated the population patterns of *A. thaliana* at different geographic scales and in various regions [e.g., [6],[18],[26],[27]]. *Arabidopsis thaliana* exhibits a range-wide pattern of isolation by distance, which can also be evident at a regional scale [e.g., [6],[18],[26],[27]], though the signal may be weaker in some parts of Europe [26]. Central Europe, including Germany, may contain a “suture zone” where genotypes from different clusters meet and mix [27],[28], making this a particularly interesting region for investigating the patterns of contemporary genetic exchange in natural populations.

In contrast to our understanding of more global patterns and the historical forces that have shaped them, we know comparatively little in *A. thaliana* about local-scale and contemporary processes such as migration and ongoing outcrossing, and how these processes might be impacted by spatial structure and environmental heterogeneity. To help fill this gap, we sampled extensively at a fine geographic scale, in a variable landscape with different patterns of human impact. We employed a large number of markers, which gave us power to detect small genetic differences and outcrossing even among closely related non-identical genotypes.

### Genetic differentiation between stands

In the Tübingen area, multi-locus genotypes showed some tendency to be more closely related to their nearest neighbors. Groups from different sub-regions were nevertheless intercalated in cluster analyses. This is consistent with previous observations of microgeographical clustering of related genotypes that does not extend to larger scales, for example in studies of local *A. thaliana* accessions from North America [21] and China [48]. These findings support the previous conclusion that individual *A. thaliana* stands are loosely connected parts of meta-populations, with some level of genetic exchange among stands occurring at local scales [e.g., [49]]. Gene flow among nearby stands and recombination within stands, even if rare, apparently suffice to cause proximal accessions to be on average more closely related than those that are further apart. Together with conclusions from other surveys [e.g., [6],[18],[26]], this points to *A. thaliana* genotypes having a discernable “local stamp” when sampled at different geographical scales, from tens of meters to thousands of kilometers. Together these results imply that local contemporary processes – such as recombination and short-range migration – and historical colonization patterns are both important factors in generating the complex spatial patterns of genetic structure observed at different scales in *A. thaliana*.

### Outcrossing rates and genetic diversity within stands

Within single contiguous stands of plants we sometimes saw evidence of extensive genetic exchange and patterns of haplotype sharing suggestive of historical recombination, in agreement with previous reports that individuals within stands are genetically closer than ones from different populations or regions [5]. In our 2007 sample, 8% of individuals were heterozygous for linked markers across parts or all of the genome, and we also found many instances of clearly recombinant, but largely or fully homozygous types. In many cases, the putative parental genotypes were also found within the same stand. Estimated effective outcrossing for the whole sample set averaged less than 2%, but varied strikingly among stands, and could be as high as 14.5%. Outcrossing of *A. thaliana* has generally been estimated to be around 1% or less [e.g., [3]–[5]], with some exceptional individual stands that had estimated rates of up to 7.5% [25]. Since *A. thaliana* has been thought to be nearly exclusively selfing, observed heterozygosity at microsatellite markers was sometimes attributed to de novo mutation rather than outcrossing [e.g., [4],[5]]. We employed genome-wide biallelic SNP markers for which this concern does not apply, since the single base mutation rate [50] is negligible compared to even a very low outcrossing rate. Furthermore, we observed heterozygosity – when present – at numerous linked markers in an individual. We are therefore confident that heterozygosity in our sample arose from outcrossing rather than de novo mutation.

Outcrossing rates calculated from *F*_IS_ values, while informative for comparisons, should be treated with caution and not necessarily be seen as reflecting the actual outcrossing rate. Other factors may also affect heterozygosity. The presence of fine-scale spatial structure together with nearest-neighbor mating can inflate homozygosity, known as the Wahlund effect [38]. Indeed, simulations have shown that the increased homozygosity, patch structure and microgeographic differentiation typical of selfing species can be generated by nearest-neighbor mating [51]. Sampled heterozygosity can also be affected by selection, when heterozygous allele combinations are advantageous. This has been observed in several self-pollinated plant species [e.g., [12],[14],[15]].

Hence in discussing outcrossing rates estimated from *F*_IS_, we can think of the calculated outcrossing as measuring “effective outcrossing” – that is, the rate of generation of heterozygous genotypes, regardless of the actual outcrossing rate of the stand in question. This borrows terminology used by Ritland where “effective selfing” is defined as “the probability that an allele chosen at random from an individual's mate is identical by descent with either allele at the same locus in that individual” [52]. Effective selfing accounts for mating with relatives due to near-neighbor mating, population structure, short dispersal distances and selection.

In our sample, at least some stands were strongly internally structured and this correlated to some degree with observed homozygosity, suggesting that the Wahlund effect [38] can contribute to homozygosity in *A. thaliana*. This implies that actual outcrossing in wild stands may exceed estimates based on marker heterozygosity. The relationship between actual outcrossing and observed heterozygosity in *A. thaliana* awaits more thorough quantification, for example by progeny array analysis of unstructured stands [e.g., [8],[53]].

In non-uniformly distributed species, self-fertilization is often associated with increased spatial genetic structure [54], but whether it is a cause or consequence of selfing is not always entirely clear. For example, species such as *A. thaliana* that require some degree of disturbance to compete successfully may exist in patchy populations because of the transience of their niche. In such situations, selfing may be selectively favored to provide reproductive assurance and mitigate the effects of small population size and unavailability of crossing partners [55]. In *A. lyrata*, an outcrossing relative of *A. thaliana* that is often patchily distributed, self-compatibility has spontaneously arisen in several populations [56],[57]. Thus, though it is clearly plausible that selfing in *A. thaliana* promotes the observed population structure, it is also conceivable that *A. thaliana* was initially patchily distributed, and selfing was selectively advantageous as a result.

### Site type and genetic diversity

It is not unusual that the genetic diversity of self-fertilizing species strongly varies among stands [e.g., [16]], and *A. thaliana* is no exception [e.g., [4],[5],[25]]. We observed *A. thaliana* growing at many different sites: some in cracks between paving stones in urban environments, others at the edges of urban gardens, along rural roadsides or in railway ballast, in grassy field borders, or in species-rich rural meadow sites. This in itself is not new: other studies have described wild *A. thaliana* stands in a range of settings [e.g., [3],[25]]. However, the correlation between site type and genetic characteristics of stands that we found, though previously hinted at [23], has not been examined and documented in detail for *A. thaliana*.

In our collection, urban stands were often small and either monotypic or contained only a few common multi-locus genotypes with little or no evidence of historical recombination among them and little or no heterozygosity. This suggests that lineages propagate in urban sites predominantly by self-fertilization or by crossing with genetically identical neighbors, and that rare migration events are likely the primary force for generating diversity in these stands. Selfing species such as *A. thaliana* can also have reduced within-population genetic diversity because of high local extinction and recolonization rates [e.g., [58]]. In the case of *A. thaliana*, whether urban stands tend to be genetically simple and homozygous because they are particularly short-lived, or because migration is so low that stands remain monotypic for extended periods, remains unknown. However, rapid local extinction has been observed in some natural *A. thaliana* populations [e.g., [21]]. Indeed, when we revisited stands that we had identified in 2007, we found *A. thaliana* grew at most sites again in 2008. However, several smaller stands, such as HaS, TüHG, TüWa and TüSB25, had disappeared.

Rural stands in our sample, in contrast to urban ones, contained many distinct, though often related genotypes. Rural stands showed stronger evidence for ancestral recombination, with extended chromosomal stretches of allele sharing in pairwise genotype comparisons, as well as extensive heterozygosity. The latter not only indicated recent outcrossing, but also likely reflected the fact that rural stands were in general less spatially structured than urban ones. These patterns suggest that rural sites may have greater long-term stability than urban ones.

Many genotypes obtained from such stands were complex mosaics of SNP identity and divergence in pairwise comparisons, while other stands were composed entirely of recombinants of just two ancestral genotypes. The intricate patterns of relatedness in these stands suggest extensive sharing of genetic information, both in the past and ongoing. This is consistent with what was observed in a smaller survey of eight stands in England, where those with low levels of human interference also had higher heterozygosity and genetic diversity than those with higher human impact [23]. A study of *A. thaliana* site ecology in Norway did not find a significant correlation between species richness and genetic diversity [25], but the stands with high diversity and some heterozygosity were also described as being from “species-rich” sites [25].

Multiple factors may contribute to the differences in observed heterozygosity between rural and urban sites. The high diversity and patterns of recombination could be an indication that rural sites are less transient than urban sites, allowing the signature of ancestral outcrossing events to survive within stands. Rural sites may also enjoy higher pollinator prevalence. Numerous pollinators, including thrips and larger flying insects such as solitary bees and dipterans, have been reported to visit flowers of *A. thaliana* in central Germany [59], and *A. thaliana* flowers may actively encourage some level of pollinator-mediated outcrossing by emitting volatiles that could serve as pollinator attractants [60]. The physical environment might affect outcrossing as well. In several self-fertilizing grasses, stands in mesic conditions showed more outcrossing than stands in xeric environments [e.g., [7],[8],[15]]. Outcrossing rates may also vary from season to season, sometimes correlating with average temperature and rainfall [e.g., [61],[62]]. We did not assay whether rural sites were in general more mesic or cooler than urban ones, but given that many rural stands were found in heavily vegetated drainage ditches, or in meadows where grasses may protect soil from drying out or shade *A. thaliana* plants, it is possible that such differences impact outcrossing.

Some rural stands with a large number of distinct genotypes were nevertheless genetically simple, with all observed types attributable to hybridization and subsequent recombination between two or three ancestral genotypes. We found such stands especially in more species-poor rural sites such as an abandoned railway platform (Ey), or an exposed slope by a rural roadside (Obn). A few other rural stands consisted of only two to three distinct haplotypes, with first-generation heterozygotes among the dominant types (Erg and Bai). We suspect that these stands were recently colonized or only recently became polymorphic due to ingress of migrants. Consistent with this, these stands were in areas disturbed by road construction activity the year prior to our collection.

By sampling in consecutive years, we found that from genetically simple sites, identical genotypes were usually recovered in the second year. For genetically more complex stands, we found numerous additional genotypes in the second year, sometimes without recovering genotypes identical to those found in the previous year. However, in many cases these distinct genotypes were closely related and clustered together with those from the previous year in the same stand. This suggests that even where additional sampling over multiple years uncovers distinct genotypes, they are for the most part drawn from a similar population sample and do not represent a completely novel array of genotypes. Some diversity could originate from persistence of seeds in the soil over several growing seasons: *A. thaliana* seeds are known to occur in soil seed banks [63],[64] where they can retain the ability to germinate for at least 30 months [65],[66]. Migration may also be a factor. Wind could distribute seeds, as could inadvertent human-mediated transport. *Arabidopsis thaliana* seeds have even been shown to germinate from rabbit dung, suggesting these animals may act as a dispersal agent [67]. In some cases the differences across years could also be due to small sample sizes relative to the actual population size and the amount of genetic diversity present in these stands.

In aggregate, our data suggest that rural stands are likely to be the primary generators of recombined genomes in *A. thaliana*, an important source of diversity via novel allele combinations. Perhaps the patterns observed in rural stands are more representative of the ancestral situation for *A. thaliana*. An ability to invade human-generated low-competition habitats may have provided open niches and new opportunities, but with the trade-off that it precipitated a shift toward higher degrees of inbreeding and reduced genetic diversity within stands.

### Summary

We have presented evidence that local-scale genotype distribution patterns in *A. thaliana* are influenced by contemporary forces such as outcrossing and site ecology, which has important implications for designing studies of natural variation and adaptation. The strong spatial differentiation and heterogeneity of local stands observed here are consistent with previous studies of *A. thaliana* [e.g., [4],[21],[22]] and of other self-fertilizing plants [e.g., [7]–[15]]. In addition, our work complements a recent study of over 5,700 plants drawn from the world-wide range of *A. thaliana* and genotyped with 139 markers [30]. Although it employed a different sampling scheme, with less detailed investigation of individual populations from the Eurasian continent, its conclusions are in broad agreement with our work.

Together with previous reports, our data suggest that patterns of isolation by distance observed at larger scales [e.g., [6],[18],[26],[27],[30]] may be generated at the local level by a combination of historical colonization and contemporary recombination among closely-spaced genotypes. Outcrossing and recombination within stands can be extensive, while gene flow between stands appears to be rare. Site type characteristics correlated with genetic patterns, and we observed enormous variation among stands in estimated outcrossing rates – from none to as high as 20%. Rural stands in species-rich meadow sites had considerably higher genetic diversity and heterozygosity than stands in more urban or species-poor sites. Rural stands are thus likely hotspots for the generation of novel allele combinations.

Effective recombination rates are sufficiently high, and effective population size sufficiently large, to break down allele associations [31],[32]. Historical recombination has been suggested as a cause for breakdown of LD in Norwegian populations [25], and may explain limited LD in other self-fertilizing species [33]. While the species-wide LD patterns are good news for genome-wide association mapping [1], an interesting opportunity is offered by the collections of naturally formed recombinant inbred lines we have identified in several stands. Recombinant inbred lines generated in the laboratory have played a major role in the analysis of natural genetic variation in *A. thaliana* [2],[68]. The recombinant genotypes we have found have survived in the wild for successive generations and thus provide a rare platform to study the ability of distinct genotypes to establish themselves in diverse habitats. With sufficiently large samples from such stands, one could monitor genotype frequencies throughout the genome in studies over multiple years to ask whether certain alleles or allele combinations are under- or overrepresented, or whether frequencies fluctuate over time as biotic and abiotic conditions change in successive years.

## Materials and Methods

### Collection and growth of plants

Seeds from individual plants were collected from 77 wild stands around Tübingen from late April to early June in 2007, and again from a subset of 21 of these stands in 2008. Seeds were germinated in growth chambers, and a single descendent individual was selected for DNA extraction.

### DNA isolation and genotyping

DNA was extracted from leaf tissue that had been frozen at −80°C using a Biosprint 96 DNA plant kit on a Biosprint 96 robotic workstation (Qiagen). SNP assays were designed as described by Warthmann and colleagues [35]. We genotyped single progeny of all 1,005 plants using 551 genome-wide single nucleotide polymorphism (SNP) markers. These included a set of 149 markers selected to optimize common variants among worldwide *A. thaliana* accessions [35], which were used on both the 2007 and 2008 samples. The 2007 samples were genotyped in addition with 402 SNP markers designed to be maximally informative between 20 world-wide accessions analyzed in a previous high-resolution SNP discovery study [69]. We culled markers with very high heterozygous call rates (suggestive of copy number variation) or high failure rates, leaving in the 2007 set a total of 436 markers, of which 431 were informative, and 133 markers in the 2008 set.

### Clustering and analysis of population genetic parameters

Population gene diversity was calculated as expected heterozygosity (*H*_e_) and as 1-Q_interindividual_, the latter was calculated in GENEPOP v. 4.0 [70]. Q_interindividual_ is the probability of identity of two alleles among individuals within a stand, estimated based on observed SNP identities. This is calculated for each marker individually, and then averaged across the genome [70]. *F*_ST_ was also calculated in GENEPOP v. 4.0, which follows the methods of Weir and Cockerham [71].

For stands of ten or more individuals, we calculated *H*_e_ and *F*_IS_ using a subsampling approach to account for variation in sample size and to allow comparisons among stands, for example among rural versus urban sites. Subsampling was performed in R [47] (scripts available on request) as follows: We took a random sample of ten individuals from each sample greater than ten and calculated *H*_e_ and *F*_IS_ for each marker. This was reiterated 100 times and average values were calculated for each marker, and then averaged across the genome to obtain the mean value for the stand. 95% confidence intervals were calculated using 1,000 iterations of Weir's bootstrapping algorithm [72]. We tested for differences between rural and urban sites with the Mann-Whitney U-test implemented in R [47] on the stand mean values for *H*_e_ and *F*_IS_ calculated from the subsampling procedure.

*F*_ST_ values for 2007 versus 2008 samples from 14 stands were similarly corrected for sampling differences using a sub-sampling approach. For each sample pair from the same site, we subsampled from the larger sample the same number of individuals as are in the smaller sample. *F*_ST_ was calculated for each sub-sample compared to the smaller sample, and this was reiterated 100 times to calculate a mean *F*_ST_ for each comparison. Confidence intervals were calculated using bootstrapping as described above.

Mantel tests for isolation by distance were performed in GENEPOP v. 4.0 [70]. Autocorrelation analyses [40] were performed in SGS [73] calculating a correlogram for Moran's I [42],[43] and a distogram for genetic distance *D_G_* [44] with pairwise comparisons grouped into 10 or 30 distance classes, with sizes 3.84 km and 0.5 km respectively. With ten distance classes, each class had 1,000 or more comparisons, while with 30 classes, each had 100 or more pairwise comparisons. 95% confidence intervals around expected mean values were calculated with 500 permutations of the data.

Pairwise genetic distance between individuals and between stands was calculated using the Maximum Likelihood procedure in MEGA 4.0 [74]. Additional statistical analyses were performed and plots and histograms generated in Kaleidagraph v.4.0.3 (Synergy Software). We scanned genotypes manually for chromosomal stretches of heterozygosity and allele identity indicative of outcrossing or historical recombination events. Outcrossing (OC) was estimated from *F*_IS_ using the standard equation: OC = 1 - ((*F*_IS_ x 2)/(1+*F*_IS_)).

We performed nonparametric clustering of the SNP data, since *A. thaliana* violates common assumptions such as free outcrossing. Nonparametric clustering was performed using nonredundant genotypes in AWClust, implemented in R [47]. AWclust was also used to calculate gap statistics to estimate cluster numbers [46],[47].

## Supporting Information

Figure S1 *H*_e_ and *F*_IS_ values calculated using a sub-sampling approach for all stands with 10 or more individuals. Error bars indicate 95% confidence intervals. Stands found in urban areas are indicated in grey, and rural sites in green.
(0.05 MB PDF)

Figure S2 Pairwise SNP differences along chromosomes. Distribution of allele differences across chromosomes. Differences in pairwise comparisons are indicated with blue diamonds, while identical genotypes are shown in yellow. Boundaries between chromosomes are indicated by vertical grey lines. Colored blocks indicate genotype identities within populations. (A) Examples of two populations with simple recombination patterns where several distinct genotypes are attributable to recombination among two multi-locus genotypes. Thus these populations are essentially natural recombinant-inbred lines. (B) An example of pairwise comparisons within a complex meadow site, showing small shared blocks among several genotypes, indicating recombination and complex resolution among a larger number of genotypes.
(0.91 MB PDF)

Figure S3 Spatial structure within stands. (A) Diagram showing sequence of unique genotypes within stands. Colors indicate identity only within stands. Grey circles denote heterozygotes with unknown parents. Half circles indicate heterozygotes with known parents color-coded. C2 is the proportion of individuals with one identical neighbor. C3 is the proportion of individuals flanked by two identical neighbors (i.e., the prevalence of clusters of three identical plants). (B) Linear regression r^2^ values for *F*_IS_ × C2 or C3 show that some homozygosity can be explained by degree of genotype.
(0.24 MB PDF)

Figure S4 Spatial autocorrelation in Tübingen accession data (see Materials and Methods). Dark blue line gives observed values while red, light blue and green denote the mean, upper bound of 95% con dence interval and lower bound of 95% con dence interval, respectively. (A) Correlogram of Moran's I statistic in 10 geographic distance classes. (B) Distogram of genetic distance in 10 geographic distance classes.
(0.23 MB PDF)

Figure S5 Histograms showing pairwise genetic distance distributions. (A) Pairwise genetic distances for comparisons of genotypes found in different stands. (B) Pairwise genetic distances of comparisons within stands.
(0.03 MB PDF)

Figure S6 Gap statistic plots generated by AWClust to infer optimal cluster number (see Materials and Methods).
(0.05 MB PDF)

Table S1 Stands sampled in the Tübingen area.
(0.09 MB PDF)

Table S2 Frequencies of distinct genotypes in each Tübingen stand.
(0.62 MB PDF)

Table S3 Diversity and outcrossing in stands with 10 or more plants.
(0.13 MB PDF)

Table S4 Identical multi-locus genotypes found in different stands.
(0.05 MB PDF)

Table S5 Genotype comparisons 2007 versus 2008.
(0.17 MB PDF)
