# Supplementary material for: Local-Scale Patterns of Genetic Variability, Outcrossing, and Spatial Structure in Natural Stands of Arabidopsis thaliana
Source: PLoS Genet. 2010 Mar 26;6(3):e1000890. doi: 10.1371/journal.pgen.1000890 (PMC2845663; doi:10.1371/journal.pgen.1000890)
Supplement: Figure S1 — H e and F IS values calculated using a sub-sampling approach for all stands with 10 or more individuals. Error bars indicate 95% confidence intervals. Stands found in urban areas are indicated in grey, and rural sites in green. (0.05 MB PDF) [file pgen.1000890.s001.pdf]

# Figure S1

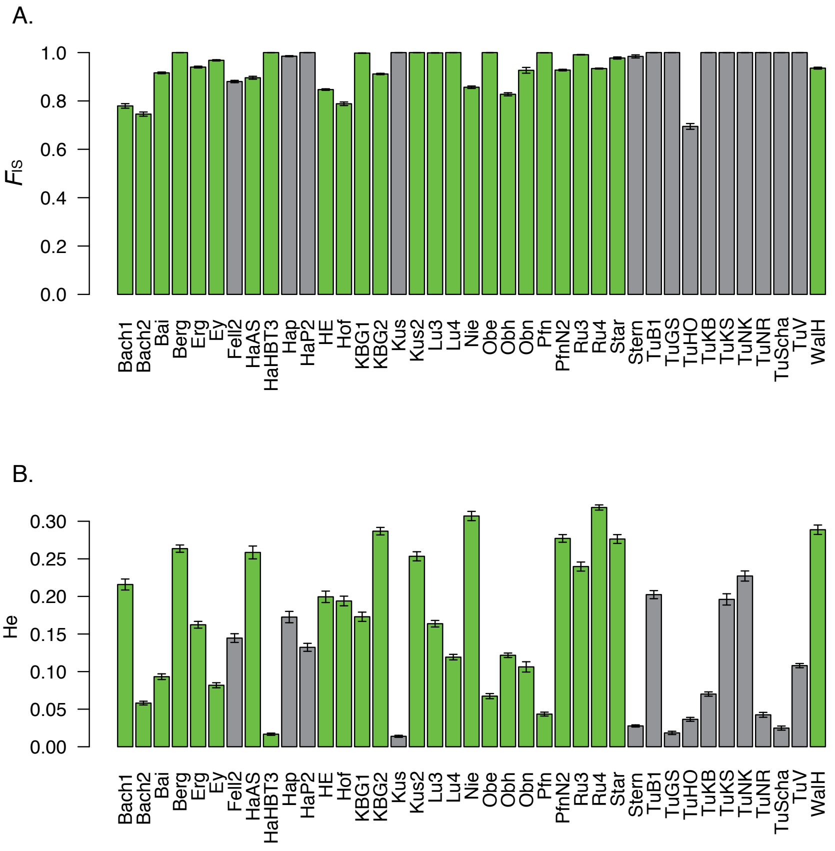


**Figure S1:** $F_{IS}$ and $H_e$ values calculated using a sub-sampling approach for all stands with 10 or more individuals. Error bars indicate 95% confidence intervals. Stands found in urban areas are indicated in grey, and rural sites in green.
