# Supplementary material for: Local-Scale Patterns of Genetic Variability, Outcrossing, and Spatial Structure in Natural Stands of Arabidopsis thaliana
Source: PLoS Genet. 2010 Mar 26;6(3):e1000890. doi: 10.1371/journal.pgen.1000890 (PMC2845663; doi:10.1371/journal.pgen.1000890)
Supplement: Figure S2 — Pairwise SNP differences along chromosomes. Distribution of allele differences across chromosomes. Differences in pairwise comparisons are indicated with blue diamonds, while identical genotypes are shown in yellow. Boundaries between chromosomes are indicated by vertical grey lines. Colored blocks indicate genotype identities within populations. (A) Examples of two populations with simple recombination patterns where several distinct genotypes are attributable to recombination among two multi-locus genotypes. Thus these populations are essentially natural recombinant-inbred lines. (B) An example of pairwise comparisons within a complex meadow site, showing small shared blocks among several genotypes, indicating recombination and complex resolution among a larger number of genotypes. (0.91 MB PDF) [file pgen.1000890.s002.pdf]

Figure S2

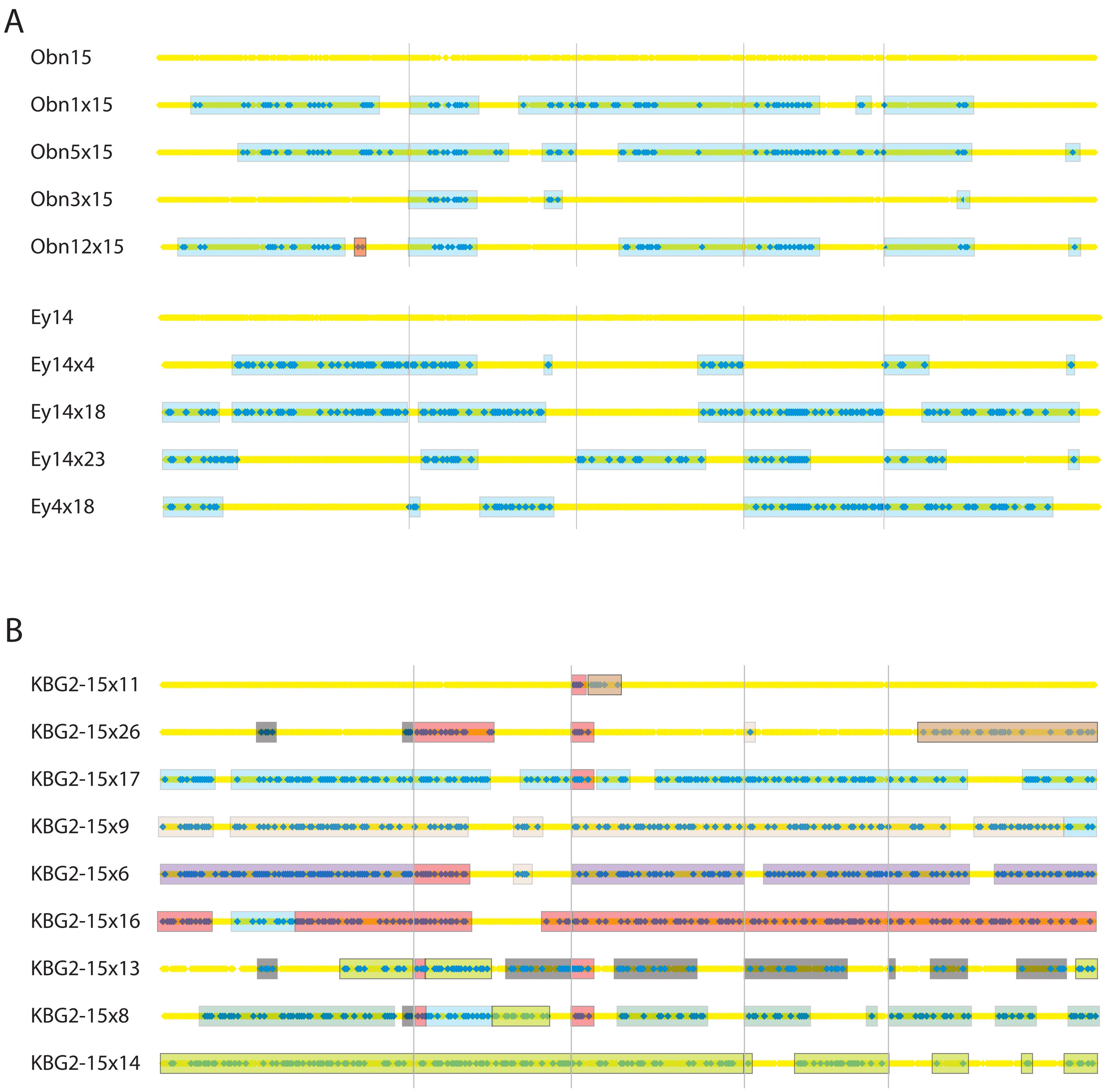

Figure S2: Distribution of allele differences across chromosomes. Differences in pairwise comparisons are indicated with blue diamonds, while identical genotypes are shown in yellow. Boundaries between chromosomes are indicated by vertical grey lines. Colored blocks indicate genotype identities within populations. (A) Examples of two populations with simple recombination patterns where several distinct genotypes are attributable to recombination among two multi-locus genotypes. Thus these populations are essentially natural recombinant-inbred lines. (B) An example of pairwise comparisons within a complex meadow site, showing small shared blocks among several genotypes, indicating recombination and complex resolution among a larger number of genotypes.
