# Supplementary material for: Local-Scale Patterns of Genetic Variability, Outcrossing, and Spatial Structure in Natural Stands of Arabidopsis thaliana
Source: PLoS Genet. 2010 Mar 26;6(3):e1000890. doi: 10.1371/journal.pgen.1000890 (PMC2845663; doi:10.1371/journal.pgen.1000890)
Supplement: Figure S3 — Spatial structure within stands. (A) Diagram showing sequence of unique genotypes within stands. Colors indicate identity only within stands. Grey circles denote heterozygotes with unknown parents. Half circles indicate heterozygotes with known parents color-coded. C2 is the proportion of individuals with one identical neighbor. C3 is the proportion of individuals flanked by two identical neighbors (i.e., the prevalence of clusters of three identical plants). (B) Linear regression r2 values for F IS × C2 or C3 show that some homozygosity can be explained by degree of genotype. (0.24 MB PDF) [file pgen.1000890.s003.pdf]

Figure S3

A

**Site**	**C2**	**C3**	
Lu4	0.900	0.567	
Schl	0.778	0.556	
Lu3	0.719	0.500	
Bai	0.645	0.226	
TuNK	0.643	0.429	
Erg	0.576	0.273	
Rü3	0.385	0.192	
HE	0.351	0.108	
WalHaes	0.333	0.095	
KBG2	0.176	0.029	
TuPK	0.125	0	*
Rü4	0.083	0.042	

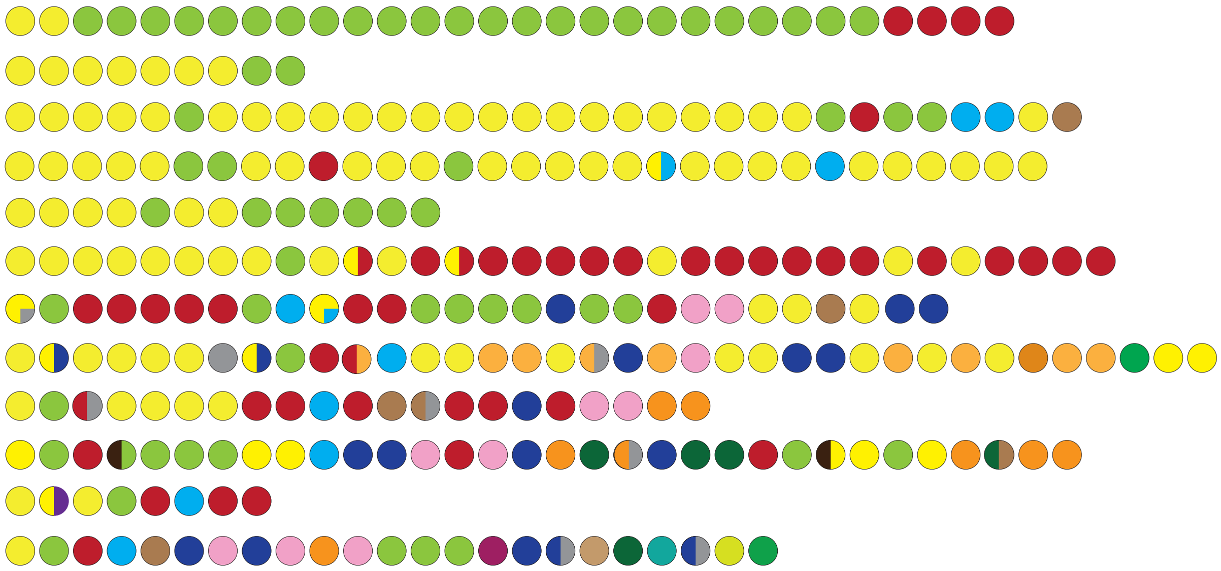

B

	Linear Regression (x Fis)		Correlation (x Fis)	
	C2	C3	C2	C3
r2	0.180	0.226	0.43	0.48

Figure S3: Spatial structure within stands.
A. Diagram showing sequence of unique genotypes within stands. Colors indicate identity only within stands. Grey circles denote heterozygotes with unknown parents. Half circles indicate heterozygotes with known parents color-coded. C2 is the proportion of individuals with one identical neighbor. C3 is the proportion of individuals flanked by two identical neighbors (i.e. the prevalence of clusters of three identical plants). B. Linear regression r2 values for F_{IS} x C2 or C3 show that some homozygosity can be explained by degree of genotype clustering. In both cases the prevalence of larger clusters is more strongly correlated with homozygosity.
