# Supplementary material for: Local-Scale Patterns of Genetic Variability, Outcrossing, and Spatial Structure in Natural Stands of Arabidopsis thaliana
Source: PLoS Genet. 2010 Mar 26;6(3):e1000890. doi: 10.1371/journal.pgen.1000890 (PMC2845663; doi:10.1371/journal.pgen.1000890)
Supplement: Figure S4 — Spatial autocorrelation in Tübingen accession data (see Materials and Methods). Dark blue line gives observed values while red, light blue and green denote the mean, upper bound of 95% con dence interval and lower bound of 95% con dence interval, respectively. (A) Correlogram of Moran's I statistic in 10 geographic distance classes. (B) Distogram of genetic distance in 10 geographic distance classes. (0.23 MB PDF) [file pgen.1000890.s004.pdf]

Figure S4

A.

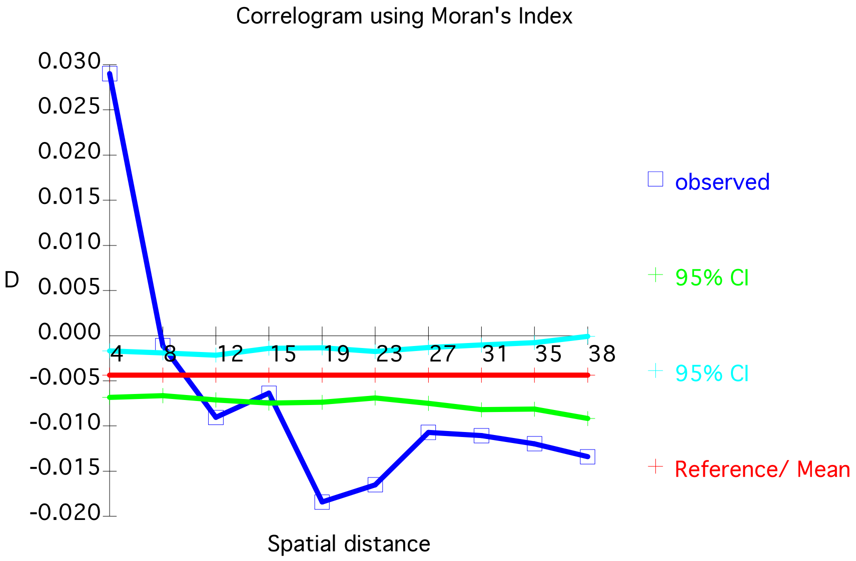

B.

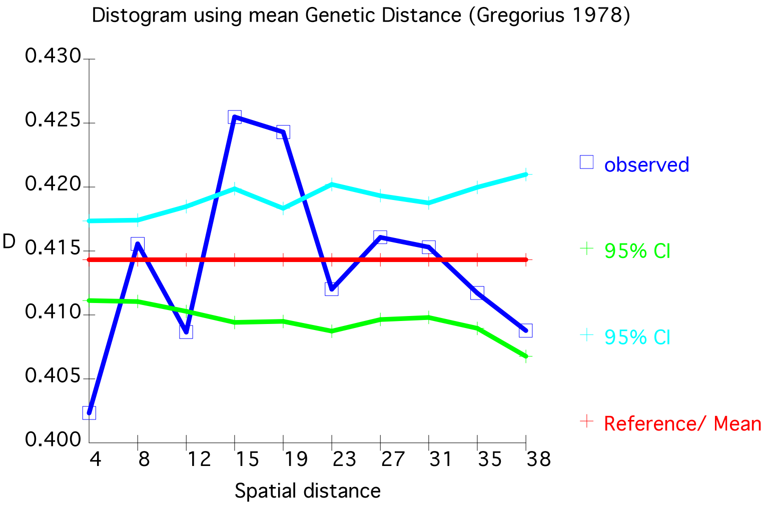

Figure S4: Spatial autocorrelation in Tübingen accession data (see materials and methods). Dark blue line gives observed values while red, light blue and green denote the mean, upper bound of 95% confidence interval and lower bound of 95% confidence interval, respectively.
A. Correlogram of Moran's I statistic in 10 geographic distance classes.
B. Distogram of genetic distance in 10 geographic distance classes.
