# Supplementary material for: Local-Scale Patterns of Genetic Variability, Outcrossing, and Spatial Structure in Natural Stands of Arabidopsis thaliana
Source: PLoS Genet. 2010 Mar 26;6(3):e1000890. doi: 10.1371/journal.pgen.1000890 (PMC2845663; doi:10.1371/journal.pgen.1000890)
Supplement: Figure S5 — Histograms showing pairwise genetic distance distributions. (A) Pairwise genetic distances for comparisons of genotypes found in different stands. (B) Pairwise genetic distances of comparisons within stands. (0.03 MB PDF) [file pgen.1000890.s005.pdf]

Figure S5

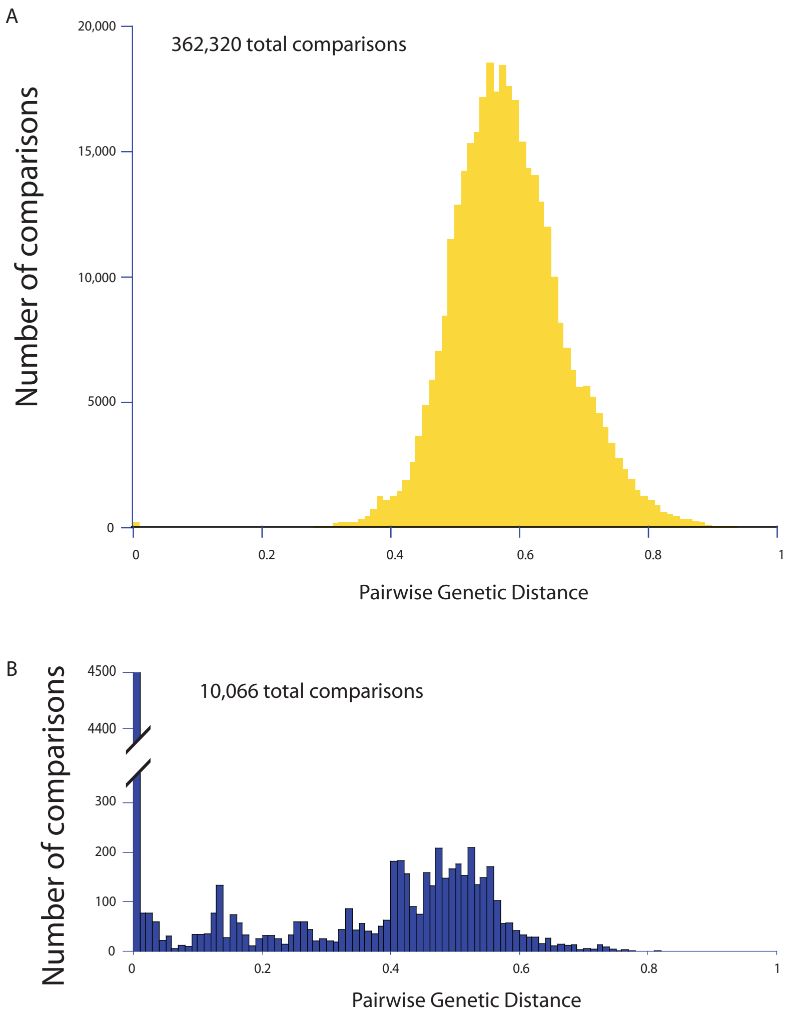

Figure S5:
Histograms showing pairwise genetic distance distributions. A. Pairwise genetic distances for comparisons of genotypes found in different stands. B. Pairwise genetic distances of comparisons within stands.
