# Supplementary material for: Local-Scale Patterns of Genetic Variability, Outcrossing, and Spatial Structure in Natural Stands of Arabidopsis thaliana
Source: PLoS Genet. 2010 Mar 26;6(3):e1000890. doi: 10.1371/journal.pgen.1000890 (PMC2845663; doi:10.1371/journal.pgen.1000890)
Supplement: Figure S6 — Gap statistic plots generated by AWClust to infer optimal cluster number (see Materials and Methods). (0.05 MB PDF) [file pgen.1000890.s006.pdf]

Figure S6

Gap statistic plots for clustering shown in Figure 2.

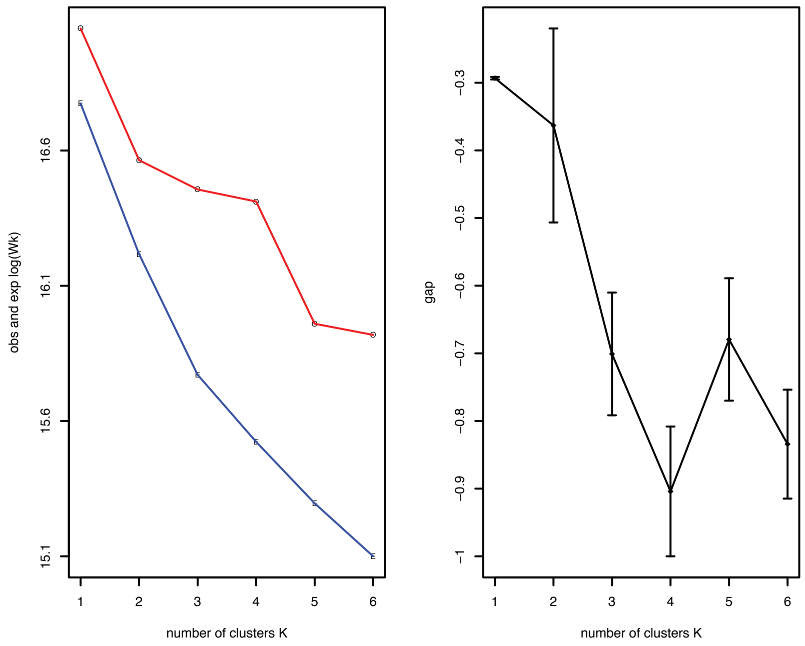

Figure S6: Gap statistic plots generated by AWClust to infer optimal cluster number (see materials and methods).
