# Supplementary material for: Local-Scale Patterns of Genetic Variability, Outcrossing, and Spatial Structure in Natural Stands of Arabidopsis thaliana
Source: PLoS Genet. 2010 Mar 26;6(3):e1000890. doi: 10.1371/journal.pgen.1000890 (PMC2845663; doi:10.1371/journal.pgen.1000890)
Supplement: Table S1 — Stands sampled in the Tübingen area. (0.09 MB PDF) [file pgen.1000890.s007.pdf]

Table S1. Stands sampled in Tübingen area.

Location	Site	Latitude (N)	Longitude (E)	N	G	H	Type[1]	Cat.[2]
Altensteig	Alt	48° 35' 36.96"	9° 13' 1.45"	7	7	1	M	Rural
Near Starzach Wachendorf	Bach	48° 24' 44.03"	8° 50' 7.96"	10	6	5	M	Rural
Near Starzach Wachendorf	Bach2	48° 24' 41.73"	8° 50' 20.24"	17	2	1	M	Rural
Baisingen	Bai	48° 30' 13.77"	8° 46' 52.57"	32	5	1	R	Rural
Near Felldorf	Berg	48° 24' 41.36"	8° 47' 35.56"	12	7	0	F	Rural
Ergenzingen	Erg	48° 29' 51.43"	8° 48' 9.51"	33	4	2	R	Rural
Bad Imnau	BI	48° 24' 12.39"	8° 46' 12.91"	4	1	0	RW	Rural
Eyach (train station)	Ey	48° 26' 46.11”	8° 46' 50.06”	40	5	1	RW	Rural
Near Felldorf	Fell1	48° 25' 13.10"	8° 47' 39.28"	5	1	0	F	Rural
Felldorf	Fell2	48° 25' 48.52"	8° 47' 17.16"	28	5	3	M	Rural
Felldorf	Fell3	48° 25' 53.60"	8° 47' 23.78"	8	7	2	R	Rural
Near Groß Engstingen	GE	48° 23' 57.08"	9° 16' 32.98"	7	1	0	R	Rural
Gniebel	Gn1	48° 34' 24.84"	9° 10' 12.21"	8	7	1	M	Rural
Häslach	Has	48° 35' 57.54"	9° 12' 12.26"	7	3	0	F	Rural
Hagelloch (Alte Steige)	HaAS	48° 32' 6.74"	9° 1' 9.78"	11	4	2	F	Rural
Hagelloch (Heuberg)	HaHBT1	48° 32' 35.40"	9° 1' 17.30"	9	4	1	M	Rural
Hagelloch (Heuberg)	HaHBT2	48° 32' 31.91"	9° 1' 8.67"	6	2	0	M	Rural
Hagelloch (Heuberg)	HaHBT3	48° 32' 34.51"	9° 1' 15.38"	16	1	0	M	Rural
Hagelloch (South)	Ha-S	48° 32' 17.15"	9° 0' 43.32"	4	2	0	F	Rural
Hagelloch (Field)	Ha-SF	48° 32' 13.43"	9° 0' 42.02"	3	1	0	F	Rural
Entringen/Hagelloch	HE	48° 33' 9.41"	8° 59' 41.38"	37	15	7	F	Rural
Höfendorf	Hof	48° 24' 23.35"	8° 50' 55.39"	21	9	5	M	Rural
Hagelloch (Kreuzberg)	KBG1	48° 31' 59.11"	9° 0' 51.44"	31	4	3	M	Rural
Hagelloch (Kreuzberg)	KBG2	48° 32' 2.42"	9° 0' 52.67"	32	14	4	M	Rural
Kusterdingen	Kus2	48° 30' 57.08"	9° 6' 36.18"	10	5	0	R	Rural
Kusterdingen (field)	Kus3	48° 30' 41.90"	9° 6' 36.14"	2	2	1	F	Rural
Pfrondorf - South	Pfn-S	48° 32' 40.73"	9° 6' 13.67"	1	1	1	F	Rural
Tübingen-Lustnau	Lu3	48° 32' 4.08"	9° 5' 12.24"	32	5	1	R	Rural
Tübingen-Lustnau	Lu4	48° 32' 14.77"	9° 5' 23.86"	30	3	0	R	Rural
Mötzingen / Bondorf	Nie	48° 31’ 24.88"	8° 48’ 58.52"	34	25	7	M	Rural
Obernau	Obe	48° 27' 15.29"	8° 52' 11.92"	14	2	0	M	Rural
Oberhausen	Obh	48° 23' 21.77"	8° 57' 22.09"	20	4	3	F	Rural
Oberndorf	Obn	48° 31' 16.77"	8° 55' 26.92"	11	8	4	R	Rural
Pfrondorf	Pfn	48° 32’ 27.94"	9° 5’ 34.10"	19	2	0	R	Rural
Near Pfrondorf	PfnN2	48° 33' 36.26"	9° 6' 33.20"	17	11	3	F	Rural
Rübgarten	Ru1	48° 33’ 45.38"	9° 9’ 24.33"	3	2	0	M	Rural
Rübgarten-north	Ru-N	48° 34’ 7.90"	9° 9’ 48.44"	3	3	0	F	Rural
Rübgarten	Ru3	48° 33’ 50.46"	9° 9’ 34.09"	28	15	4	M	Rural
Near Rübgarten	Ru4	48° 34’ 24.19"	9° 9’ 47.96"	24	17	4	M	Rural
Schlaitdorf	Schl	48° 36’ 13.12"	9° 13’ 1.28"	9	2	0	R	Rural
Near Starzach Bierlingen	Star	48° 25’ 4.52"	8° 49’ 0.18"	11	9	2	F	Rural
Tübingen (North)	TüWH	48° 32' 47.36"	9° 3' 47.36"	1	1	0	M	Rural

Walddorf-Häslach	WalHäs	48° 35’ 44.00"	9° 11’ 7.91"	21	10	2	F	Rural
Wankheim	Wank	48° 29' 45.87"	9° 6' 37.08"	5	3	0	F	Rural
Bebenhausen-Alte Straße	Bbn-AS	48° 33' 32.71"	9° 3’ 38.92"	4	1	0	PS	Urban
Bebenhausen-Kloister	Bbn-K	48° 33’ 40.53"	9° 3’ 40.58"	3	1	0	PS	Urban
Bodelshausen	Bod	48° 23' 31.55"	8° 57' 56.39"	6	1	0	PS	Urban
Bondorf	Bon	48° 31' 9.19"	8° 50' 31.35"	9	1	0	R	Urban
Gniebel	Gn2	48° 34' 34.10"	9° 10' 55.42"	6	3	0	PS	Urban
Hagelloch	Ha3	48° 32' 20.77"	9° 0' 38.00"	7	1	0	PS	Urban
Hagelloch	HaP	48° 32' 22.66"	9° 0' 31.09"	14	4	1	PS	Urban
Hagelloch	HaP2	48° 32' 20.49"	9° 0' 36.39"	10	2	0	PS	Urban
Hagelloch (Sport field)	Ha-SP	48° 32' 20.11"	9° 0’ 31.64"	2	2	0	PS	Urban
Hart	Hart	48° 23’ 11.57"	8° 50’ 53.95"	3	2	1	Gn	Urban
Hirschau	HI	48° 29’ 48.35"	8° 59’ 48.90"	5	4	0	PS	Urban
Kusterdingen	Kus	48° 31' 17.70"	9° 6' 35.76"	26	1	0	PS	Urban
Pfrondorf-North	Pfn-N1	48° 32’ 50.05"	9° 6’ 15.94"	2	2	0	Gn	Urban
Tübingen-Lustnau	Lu1	48° 31' 51.45"	9° 4’ 51.59"	3	1	0	PS	Urban
Tübingen-Lustnau	Lu2	48° 31' 49.95"	9° 5' 1.05"	8	1	0	PS	Urban
Bad Imnau - Mühringen	Muh	48° 25' 10.36"	8° 45' 35.04"	9	2	1	RW	Urban
Tübingen (North)	Stern	48° 32’ 17.91"	9° 3’ 17.93"	24	1	1	Gn	Urban
Tübingen (East)	TüB1	48° 31’ 5.97"	9° 4’ 30.10"	16	3	0	PS	Urban
Tübingen (East)	TüB2	48° 31' 10.48"	9° 4’ 41.25"	8	2	0	PS	Urban
Tübingen (central)	TüGS	48° 31’ 7.47"	9° 3’ 47.88"	10	1	0	PS	Urban
Tübingen (central)	TüHG	48° 31’ 14.47"	9° 2’ 58.05"	3	1	0	PS	Urban
Tübingen (North)	TüHO	48° 32' 20.01"	9° 3' 14.80"	20	1	1	PS	Urban
Tübingen (central)	TüHT	48° 31’ 10.83"	9° 3’ 9.31"	3	2	0	PS	Urban
Tübingen (central)	TüKB	48° 31' 16.68"	9° 3' 7.52"	21	2	0	PS	Urban
Tübingen (central)	TüKS	48° 31’ 32.31"	9° 3’ 54.50"	14	2	0	PS	Urban
Tübingen (central)	TüNK	48° 31' 28.21"	9° 3' 6.76"	13	2	0	PS	Urban
Tübingen (North)	TüNR	48° 32’ 23.04"	9° 3’ 51.36"	11	1	0	PS	Urban
Tübingen (central)	TüPK	48° 31' 25.54"	9° 3' 9.25"	8	5	1	Gn	Urban
Tübingen (North)	TüScha	48° 32' 3.98"	9° 3' 11.96"	23	1	0	PS	Urban
Tübingen (central)	TüV	48° 31’ 23.62"	9° 3’ 7.12"	10	2	0	PS	Urban
Tübingen (West)	TüW1	48° 31’ 29.51"	9° 1’ 31.71"	7	2	0	PS	Urban
Tübingen (North)	TüWa	48° 32' 30.81"	9° 2’ 42.40"	2	1	0	PS	Urban
Wendelsheim	Wen	48° 30' 24.52"	8° 56' 11.31"	9	1	0	PS	Urban

Notes:
N = number of individuals sampled. Where N<20, all individuals in the stand were sampled.
G = Number of distinct whole-genome genotypes identified within stand.
H = Number of partly or fully heterozygous individuals in population.
[1] Type of site where F = Fieldside, Gn = Garden, M = Meadow, PS = Paving stones, R = Roadside, RW = Railway
[2] Site type category where “Rural” indicates sites in areas of lower human impact during the *A. thaliana* growing season such as meadows or field borders, while “Urban” indicates sites located in towns with high human influence such as parking lots, sidewalks or urban gardens.
