# Supplementary material for: Local-Scale Patterns of Genetic Variability, Outcrossing, and Spatial Structure in Natural Stands of Arabidopsis thaliana
Source: PLoS Genet. 2010 Mar 26;6(3):e1000890. doi: 10.1371/journal.pgen.1000890 (PMC2845663; doi:10.1371/journal.pgen.1000890)
Supplement: Table S2 — Frequencies of distinct genotypes in each Tübingen stand. (0.62 MB PDF) [file pgen.1000890.s008.pdf]

Table S2. Frequencies of distinct genotypes in each Tübingen stand.

Site	Cat.	N	G	H	Genotype frequencies (excluding highly heterozygous individuals)[1]																								
Alt	Rural	7	7	1	1	1	1	1	1	1	1																		
Bach	Rural	10	6	5	2	2	1	1	1	1																			
Bach2	Rural	17	2	1	15	1																							
Bai	Rural	32	5	1	26	3	1	1																					
Berg	Rural	12	7	0	6	1	1	1	1	1	1																		
Erg	Rural	33	4	2	17	13	2	1																					
Ey	Rural	40	5	1	29	7	2	1																					
Fell	Rural	5	1	0	5																								
Fell2	Rural	28	5	3	20	4	1	1																					
Fell3	Rural	8	7	2	2	1	1	1	1	1	1																		
GE	Rural	7	1	0	7																								
Gn1	Rural	7	7	1	1	1	1	1	1	1	1																		
HaAS	Rural	11	4	2	4	4	1	1																					
HaHBT*	Rural	35	4	1	24	6	3	1																					
HE	Rural	37	15	7	14	8	2	2	1	1	1	1	1	1	1	1	1	1	1										
Hof	Rural	21	9	5	8	6	1	1	1	1	1	1	1																
KBG1	Rural	31	4	0	19	7	4	1																					
KBG2	Rural	32	14	4	6	4	4	4	2	2	1	1	1	1	1	1	1	1											
Kus2	Rural	10	5	0	4	3	1	1	1																				
Lu3	Rural	32	5	0	24	3	2	2	1																				
Lu4	Rural	30	3	0	24	4	2																						
Nie	Rural	34	25	7	4	3	2	2	2	2	1	1	1	1	1	1	1	1	1	1	1	1	1	1	1	1	1	1	1
Obe	Rural	14	2	0	13	1																							
Obh	Rural	20	4	3	17	1	1	1																					
Obn	Rural	11	8	3	3	2	1	1	1	1	1	1																	
Pfn	Rural	19	2	0	18	1																							
PfnN2	Rural	17	11	3	5	2	2	1	1	1	1	1	1	1	1														
Ru3	Rural	28	15	2	8	7	2	2	2	1	1	1	1	1	1	1	1	1	1										

Ru4	Rural	24	17	4	5	3	3	1	1	1	1	1	1	1	1	1	1	1	1	1	1
Schl	Rural	9	2	0	7	2															
Star	Rural	11	9	2	3	1	1	1	1	1	1	1	1								
WalHäs	Rural	21	10	2	6	5	2	2	1	1	1	1	1	1							
Wank	Rural	5	3	0	3	1	1														
Bod	Urban	6	1	0	6																
Bon	Urban	9	1	0	9																
Gn2	Urban	6	3	0	3	2	1														
Ha3	Urban	7	1	0	7																
HaP	Urban	14	4	1	8	5	1														
HaP2	Urban	10	2	0	7	3															
Kus	Urban	26	1	0	26																
Lu2	Urban	8	1	0	8																
Muh	Urban	9	3	0	7	2															
Stern	Urban	24	1	1	24																
TüB1	Urban	16	3	0	10	5	1														
TüB2	Urban	8	2	0	7	1															
TüGS	Urban	10	1	0	10																
TüHO	Urban	20	1	0	20																
TüKB	Urban	21	2	0	20	1															
TüKS	Urban	14	2	0	8	6															
TüNK	Urban	13	2	0	7	6															
TüNR	Urban	11	1	0	11																
TüPK	Urban	8	5	1	3	2	1	1	1												
TüScha	Urban	23	1	0	23																
TüV	Urban	10	2	0	9	1															
TüW1	Urban	7	2	0	6	1															
Wen	Urban	9	1	0	9																

*Data from three populations that were very close to each other combined.

Notes: N = individuals genotyped, G = multi-locus genotypes differing by at least one SNP, H = partly or fully heterozygous individuals.

1. Highly heterozygous individuals, which could not be unambiguously assigned as separate “types” are not included in the genotype frequency counts. Thus in some populations, “genotype frequencies” do not sum to the total number of plants sampled.
