# Supplementary material for: Local-Scale Patterns of Genetic Variability, Outcrossing, and Spatial Structure in Natural Stands of Arabidopsis thaliana
Source: PLoS Genet. 2010 Mar 26;6(3):e1000890. doi: 10.1371/journal.pgen.1000890 (PMC2845663; doi:10.1371/journal.pgen.1000890)
Supplement: Table S3 — Diversity and outcrossing in stands with 10 or more plants. (0.13 MB PDF) [file pgen.1000890.s009.pdf]

Table S3. Diversity and outcrossing in stands with 10 or more plants.

Site	N	1-Q[1]	H_e[2]	CI[3]	F_{IS}[4]	CI[3]	EOC[5]	CI[3]	Cat.[6]
Bach	10	0.230	0.216	0.209-0.223	0.779	0.770-0.789	12.4	11.8-13.1	Rural
Bach2	17	0.057	0.058	0.056-0.061	0.746	0.737-0.754	14.5	19.3	Rural
Bai	32	0.093	0.093	0.089-0.097	0.916	0.913-0.920	4.4	4.2-4.6	Rural
Berg	12	0.284	0.264	0.259-0.268	1		0		Rural
Erg	33	0.167	0.162	0.158-0.167	0.940	0.936-0.944	3.1	2.9-3.3	Rural
Ey	40	0.073	0.082	0.078-0.085	0.968	0.965-0.970	1.6	1.5-1.8	Rural
Fell2	28	0.142	0.145	0.139-0.150	0.880	0.875-0.884	6.4	6.1-6.6	Rural
HaAS	11	0.283	0.259	0.250-0.267	0.896	0.890-0.902	5.5	5.2-5.8	Rural
HaHBT3	16	0.001	0.017	0.015-0.018					Rural
HE	37	0.210	0.199	0.192-0.207	0.847	0.844-0.850	8.3	8.1-8.5	Rural
Hof	21	0.208	0.194	0.188-0.200	0.788	0.781-0.796	11.8	11.4-12.3	Rural
KBG1	31	0.183	0.173	0.167-0.179	0.998	0.997-0.999	0.1	0.1-0.2	Rural
KBG2	32	0.314	0.287	0.282-0.292	0.911	0.909-0.914	4.6	4.5-4.8	Rural
Kus2	10	0.267	0.253	0.247-0.259	1		0		Rural
Lu3	32	0.151	0.164	0.159-0.168	0.999	0.998-0.999	0.1	0-0.1	Rural
Lu4	30	0.119	0.119	0.116-0.123	1		0		Rural
Nie	34	0.339	0.307	0.301-0.313	0.857	0.852-0.862	7.7	7.4-8.0	Rural
Obe	14	0.059	0.067	0.064-0.071	1		0		Rural
Obh	20	0.118	0.122	0.119-0.125	0.827	0.821-0.834	9.4	9.1-9.8	Rural
Obn	11	0.101	0.106	0.099-0.113	0.927	0.915-0.938	3.8	3.2-4.5	Rural
Pfn	19	0.040	0.043	0.041-0.046	0.999	0.999-1.000	0	0-0.1	Rural
PfnN2	17	0.307	0.277	0.272-0.282	0.928	0.924-0.931	3.8	3.6-3.9	Rural
Ru3	28	0.255	0.240	0.233-0.246	0.991	0.990-0.992	0.4	0.4-0.5	Rural
Ru4	24	0.352	0.318	0.315-0.322	0.933	0.932-0.936	3.4	3.3-3.5	Rural
Star	11	0.298	0.276	0.271-0.283	0.977	0.973-0.982	1.1	0.9-1.4	Rural
WalHäs	21	0.315	0.289	0.282-0.295	0.936	0.932-0.939	3.3	3.1-3.5	Rural
HaP	14	0.177	0.173	0.165-0.180	0.985	0.983-0.987	0.8	0.6-0.9	Urban
HaP2	10	0.124	0.132	0.127-0.138	1		0		Urban
Kus	26	0	0.014	0.013-0.015					Urban
Stern	24	0.002	0.028	0.026-0.029	0.984	0.978-0.990	0.8	0.5-1.13	Urban
TüB1	16	0.217	0.202	0.197-0.208	1		0		Urban
TüGS	10	0.001	0.018	0.016-0.021					Urban
TüHO	20	0.002	0.036	0.034-0.039	(0.695)	(0.683-0.706)	(18.0)	(17.2-18.9)	Urban
TüKB	21	0.045	0.070	0.067-0.073	1		0		Urban
TüKS	14	0.200	0.196	0.189-0.204	1		0		Urban
TüNK	13	0.241	0.227	0.220-0.234	1		0		Urban
TüNR	11	0.006	0.043	0.039-0.046					Urban
TüScha	23	0	0.025	0.022-0.028					Urban
TüV	10	0.102	0.108	0.105-0.111	1		0		Urban

Notes:

[1] 1-Q is the inter-individual, intra-population diversity calculated in Genepop (See materials and methods). It was calculated only for sites with five or more individuals.

[2] H_e = Expected heterozygosity from Hardy-Weinberg calculated using sub-sampling to compensate for variation in sample size (see materials and methods).
[3] 95% confidence interval.
[4] F_{is} was calculated only for stands with more than five individuals and more than one genotype (or heterozygotes) present. Numbers for TuHO are given in parentheses because they are based on a single heterozygous individual in an otherwise monotypic stand.
[5] Effective Outcrossing (EOC) estimated from F_{is} as: EOC = 1 - ((F_{is} x 2) / (1+F_{is})); Numbers for TuHO are given in parentheses because they are based on a single heterozygous individual in an otherwise monotypic stand.
[6] Site type category where “Rural” indicates sites in areas of lower human impact during the *A. thaliana* growing season such as meadows or field borders, while “Urban” indicates sites located in towns with high human influence such as parking lots, sidewalks or urban gardens.
