# Supplementary material for: Local-Scale Patterns of Genetic Variability, Outcrossing, and Spatial Structure in Natural Stands of Arabidopsis thaliana
Source: PLoS Genet. 2010 Mar 26;6(3):e1000890. doi: 10.1371/journal.pgen.1000890 (PMC2845663; doi:10.1371/journal.pgen.1000890)
Supplement: Table S4 — Identical multi-locus genotypes found in different stands. (0.05 MB PDF) [file pgen.1000890.s010.pdf]

Table S4. Identical multi-locus genotypes found in different Tübingen stands.

Site 1		Site 2		Inter-stand
**Stand**	**Prevalence**†	**Stand**	**Prevalence**†	**distance (km)**
RüN	1 / 3	Rü4	1 / 24	0.50
TüPK	3 / 8	TüV	1 / 10	0.07
TüKB	20 / 21	TüV	9 / 10	0.22
TüGS	10 / 10	TüKB	1 / 21	1.08
TüHG	3 / 3	TüHT	1 / 3	0.25
HaHBT1	4 / 9	HaHBT2	2 / 6	0.21
HaHBT1	4 / 9	HaHBT2	4 / 6	0.21
HaHBT1	4 / 9	HaHBT3	16 / 16	0.05
TüB1	10 / 16	TüB2	7 / 8	0.27
TüKS	6 / 14	TüSB57	4 / 4	1.17
HaAS	5 / 11	HaSB	1 / 7	0.63
Erg	1 / 33	Fell3	1 / 8	7.40
GE	7 / 7	TüPK	2 / 8	21.00
Nie	2 / 34	Obh	1 / 20	18.00
Nie	4 / 34	Pfn	1 / 18	21.00

†proportion of shared genotype among all individuals in that stand.
