# Supplementary material for: Local-Scale Patterns of Genetic Variability, Outcrossing, and Spatial Structure in Natural Stands of Arabidopsis thaliana
Source: PLoS Genet. 2010 Mar 26;6(3):e1000890. doi: 10.1371/journal.pgen.1000890 (PMC2845663; doi:10.1371/journal.pgen.1000890)
Supplement: Table S5 — Genotype comparisons 2007 versus 2008. (0.17 MB PDF) [file pgen.1000890.s011.pdf]

Table S5. Comparison of multi-locus genotype frequencies in Tübingen area in 2007 vs. 2008.

Site	Year	N	Distinct multi-locus genotypes: All	Shared	Distinct	% distinct	hets	PW F_{st}[1]	Frequency of genotypes[2]
Bl	2007	4	1	4	0	0			4
	2008	8	1	8	0	0			8
BbnAS	2007	4	1	4	0	0			4
	2008	3	1	3	0	0			3
Gn	2007	3	1	3	0	0			3
	2008	7	1	7	0	0			7
Lu1	2007	3	1	3	0	0			3
	2008	2	1	2	0	0			2
TüHO	2007	21	1	21	0	0			21
	2008	4	1	4	0	0			4
TüNR	2007	11	1	11	0	0			11
	2008	8	1	8	0	0			8
Wen	2007	9	1	9	0	0		0.032	9
	2008	9	2	8	1	12		±0.0002	8 1
Obe	2007	14	2	14	0	0		0.085	13 1
	2008	20	2	20	0	0		±0.0005	15 5
Bai	2007	32	3	26	5	16	1	0.062	26 4 1
	2008	15	1	15	0	0		±0.0004	15
Bach2	2007	17	3	16	0	0	1	0.047	15 1
	2008	26	7	16	10	35	2	±0.0003	15 1 6 3 1
Erg	2007	33	4	13	18	55	2	0.051	17 13 1
	2008	6	1	6	0	0		±0.0003	6
Ey	2007	40	5	29	10	23	1	0.072	29 7 2 1
	2008	41	5	20	23	56		±0.0004	20 16 3 3 1
Fell2	2007	26	6	20	6	23	2	0.073	20 4 1 1
	2008	10	1	10	0	0		±0.0005	10
Berg	2007	12	7	0	12	100		0.030	6 1 1 1 1 1 1
	2008	9	8	0	9	100	1	±0.0002	2 1 1 1 1 1 1
Fell3	2007	9	7	0	8	100	1	0.050	2 1 1 1 1 1 1
	2008	11	8	0	11	100		±0.0003	4 1 1 1 1 1 1 1
Gn1	2007	8	7	1	6	86	1	0.074	2 1 1 1 1 1

	2008	11	8	2	8	80	1	±0.0005	2 2 2 1 1 1 1
Bach1	2007	12	7	3	7	70	2	0.038	2 2 2 1 1 1 1
	2008	10	10	2	5	71	3	±0.0002	1 1 1 1 1 1 1
PfnN2	2007	16	10	0	14	100	2	0.033	5 2 2 1 1 1 1 1
	2008	11	10	0	8	100	3	±0.0002	2 1 1 1 1 1 1
Rü4	2007	25	17	3	19	86	2	0.066	4 3 3 1 1 1 1 1 1 1 1 1 1 1 1
	2008	27	13	8	16	67	3	±0.0004	2 2 4 8 3 1 1 1 1 1
Nie	2007	32	25	3	24	89	7	0.129	4 3 2 2 2 2 1 1 1 1 1 1 1 1 1 1 1 1
	2008	50	16	5	39	89	7	±0.001	1 1 3 20 4 3 2 2 1 1 1 1 1 1 1 1

[1]F_{ST} values comparing 2007 and 2008 genotype sets found within each site. For comparison, average Fst among all sites (pairwise comparisons) is 0.51. F_{ST} was calculated using a subsampling approach to correct for sample size differences between years. ± indicates 95% confidence interval calculated from subsampling.

[2]Prevalences of unique genotypes in each population and each year. Green indicates genotypes identical in both years, yellow indicates genotypes unique to 2007, orange indicates genotypes unique to 2008.
